# Encoding scheme using quantum dots for single logical qubit information onto four-photon decoherence-free states

**DOI:** 10.1038/s41598-020-71072-0

**Published:** 2020-09-18

**Authors:** Jino Heo, Changho Hong, Min-Sung Kang, Hyung-Jin Yang

**Affiliations:** 1 Institute of Natural Science, Korea University, Sejong, 30091 Republic of Korea; 2 The Affiliated Institute of Electronics and Telecommunications Research Institute, P.O. Box 1, Yuseong, Daejeon, 34188 Republic of Korea; 3 Korean Intellectual Property Office (KIPO), Government Complex Daejeon Building 4, 189, Cheongsa-ro, Seo-gu, Daejeon, 35208 Republic of Korea; 4 Department of Physics, Korea University, Sejong, 30091 Republic of Korea

**Keywords:** Quantum optics, Quantum information

## Abstract

We designed an encoding scheme, using quantum dots (QDs), for single logical qubit information by encoding quantum information onto four-photon decoherence-free states to acquire immunity against collective decoherence. The designed scheme comprised of QDs, confined in single-sided cavities (QD-cavity systems), used for arbitrary quantum information, encoded onto four-photon decoherence-free states (logical qubits). For our scheme, which can generate the four-photon decoherence-free states, and can encode quantum information onto logical qubits, high efficiency and reliable performance of the interaction between the photons and QD-cavity systems is essential. Thus, through our analysis of the performance of QD-cavity systems under vacuum noise and sideband leakage, we demonstrate that the encoding scheme for single logical qubit information could be feasibly implemented.

## Introduction

Quantum phenomena can produce various quantum information processing schemes, such as quantum communications^1–4^, quantum computation^5–11^, quantum controlled operations^12–15^, and quantum entanglement^16–22^, in theory and practice. And if these schemes are experimentally realized, the mitigation of the decoherence effect will be a pivotal issue for the reliable quantum information processing. Owing to the influence of decoherence induced by uncontrolled interactions between systems and the environment, an uncontrolled non-unitary process inevitably occurs during quantum information processing. Therefore, to compensate for the decoherence effect, two methods have been researched: active processes with reference frames and passive processes without reference frames. Active processes include quantum error correction codes^23–25^, dynamic decoupling controls^26–28^, and feedback controls^29–31^.

On the other hand, when collective decoherence, dephasing noise and rotation noise, occurs in the transmission of photons, the active process with reference frames cannot correct the transferred photons. Collective decoherence^32–34^ means that each qubit carrier in the subsystem undergoes the effect induced by the identical decoherence. As an example of collective decoherence, the collective rotation noise, ${\text{U}}_{{\text{R}}}$, affect polarizations of photon (i.e. ${\text{U}}_{{\text{R}}} \left| H \right\rangle \to \, \cos \theta \left| R \right\rangle + {\sin}\theta \left| L \right\rangle$ and ${\text{U}}_{{\text{R}}} \left| V \right\rangle \to \, - \sin \theta \left| R \right\rangle + {\cos}\theta \left| L \right\rangle$ where the error is represented by ${\theta }$). In the passive processes, the methods of decoherence-free subspaces^32–34^ can be utilized to prevent collective decoherence. Because the interaction, between the system and the environment, under the collective decoherence shows a symmetry, the subspaces of decoherence-free states can be immune against the decoherence induced by the symmetrical interaction. When the decoherence-free states are influenced by this symmetrical interaction, they exhibit some symmetry, despite the strong interaction between qubit and environment. Thus, the quantum information in decoherence-free states is invariant under collective decoherence. A simple method to reduce the affection of collective decoherence is to encode the quantum information onto a two-qubit decoherence-free state, as a singlet state^35^. However, for the efficient protection of quantum information, the minimum requirement for the decoherence-free subsystem is the dimension of the above two-qubit physical system. Thus, many schemes, which are capable of encoding quantum information onto three-qubit systems, have been proposed, such as the entangled *W* state^36–42^ and three-qubit decoherence-free state^21,22,43–46^. But, utilizations of two-qubit^35^ or three-qubit^21,22,43–46^ can also provide only minimal effect for the preservation of quantum information under the affections of noise in quantum channel. Thus, the applications of a four-qubit decoherence-free subspace have been designed for increasing the maintenance of coherent quantum information. For the generations of four-qubit decoherence-free state, many researchers have exploited various resources, such as spontaneous parametric down conversions (SPDC)^46,47^ or source of entangled state^48,49^ with linear optics, Zeno-like measurements and post-selections^50^, cavity-QED^44,51^, and cross-Kerr nonlinearities (XKNLs)^52,53^.

In this paper, we propose an encoding scheme assisted by quantum dots (QDs) for single logical qubit information by encoding arbitrary quantum information onto four-photon decoherence-free states to prevent collective decoherence^32–34^. In our scheme, the QD-cavity (single-sided) systems, which can interact between a flying photon (photonic qubit) and an excess electron (spin qubit) of the QD, are crucial elements for the realization of single logical qubit information encoded arbitrary quantum information and the generation of four-photon decoherence-free states. Thus, for a reliable performance of the QD-cavity systems interactions, we quantify the experimental conditions of a QD-cavity system under vacuum noise in QD-dipole operation, and leaky modes (sideband leakage and absorption) in cavity mode^11,20,22,54–58^ via analysis of the Heisenberg equation of motion^57^. Consequently, we demonstrate that the encoding scheme for single logical qubit information onto four-photon decoherence-free states is robust against collective decoherence and experimental feasibility.

## Optical scheme to generate four-photon decoherence-free states and encode single logical qubit information

### The fundamental concept of four-photon decoherence-free states and single logical qubit information

For robust quantum information processing against collective decoherence, due to uncontrolled coupling between a system and the environment, the utilization of logical qubits $\left\{ {\left| {0_{{\text{ L}}} } \right\rangle , \, \left| {1_{{\text{ L}}} } \right\rangle } \right\}$ based on a decoherence-free subspace has been proposed^32–34^. Thus, one of the proposed concepts^32–35,43–45,50–52,59–61^ for logical qubits is the design of four-qubit decoherence-free states^44,50–52^, as follows:

1 $$\begin{aligned} \left| {0_{{\text{ L}}} } \right\rangle_{1234} & \equiv \frac{1}{2}\left( {\left| {0101} \right\rangle - \left| {0110} \right\rangle - \left| {1001} \right\rangle + \left| {1010} \right\rangle } \right)_{1234} \\\, &= \frac{1}{\sqrt 2 }\left( {\left| {01} \right\rangle - \left| {10} \right\rangle } \right)_{12} \otimes \frac{1}{\sqrt 2 }\left( {\left| {01} \right\rangle - \left| {10} \right\rangle } \right)_{34} , \\ \left| {1_{{\text{ L}}} } \right\rangle_{1234} & \equiv \frac{1}{{\sqrt {12} }}\left( {2\left| {0011} \right\rangle + 2\left| {1100} \right\rangle - \left| {0101} \right\rangle - \left| {1010} \right\rangle - \left| {0110} \right\rangle - \left| {1001} \right\rangle } \right)_{1234} \\ \, & = \frac{1}{\sqrt 3 }\left[ {\left( {\left| {0011} \right\rangle + \left| {1100} \right\rangle } \right)_{1234} - \frac{1}{\sqrt 2 }\left( {\left| {01} \right\rangle + \left| {10} \right\rangle } \right)_{12} \otimes \frac{1}{\sqrt 2 }\left( {\left| {01} \right\rangle + \left| {10} \right\rangle } \right)_{34} } \right]. \\ \end{aligned}$$

Furthermore, in quantum information processing technologies, a flying photon is a feasible resource to manipulate, transfer, and encode quantum information. Four-photon decoherence-free states $\left\{ {\left| {0_{{\text{ PL}}} } \right\rangle , \, \left| {1_{{\text{ PL}}} } \right\rangle } \right\}$, which consist of photonic spins, photons, can be used as logical qubits, $\left\{ {\left| {0_{{\text{ L}}} } \right\rangle , \, \left| {1_{{\text{ L}}} } \right\rangle } \right\}$, to carry quantum information under collective decoherence, as follows:

2 $$\begin{aligned} \left| {0_{{\text{ PL}}} } \right\rangle_{{{\text{ABCD}}}} & \equiv \frac{1}{2}\left( {\left| {RLRL} \right\rangle - \left| {RLLR} \right\rangle - \left| {LRRL} \right\rangle + \left| {LRLR} \right\rangle } \right)_{{{\text{ABCD}}}}\\ \, & = \frac{1}{\sqrt 2 }\left( {\left| {RL} \right\rangle - \left| {LR} \right\rangle } \right)_{{{\text{AB}}}} \otimes \frac{1}{\sqrt 2 }\left( {\left| {RL} \right\rangle - \left| {LR} \right\rangle } \right)_{{{\text{CD}}}} , \, \\ \left| {1_{{\text{ PL}}} } \right\rangle_{{{\text{ABCD}}}} & \equiv \frac{1}{{\sqrt {12} }}\left( {2\left| {RRLL} \right\rangle + 2\left| {LLRR} \right\rangle - \left| {RLRL} \right\rangle - \left| {LRLR} \right\rangle - \left| {RLLR} \right\rangle - \left| {LRRL} \right\rangle } \right)_{{{\text{ABCD}}}} \\ \, & = \frac{1}{\sqrt 3 }\left[ {\left( {\left| {RRLL} \right\rangle + \left| {LLRR} \right\rangle } \right)_{{{\text{ABCD}}}} - \frac{1}{\sqrt 2 }\left( {\left| {RL} \right\rangle + \left| {LR} \right\rangle } \right)_{{{\text{AB}}}} \otimes \frac{1}{\sqrt 2 }\left( {\left| {RL} \right\rangle + \left| {LR} \right\rangle } \right)_{{{\text{CD}}}} } \right], \\ \end{aligned}$$

where $\left\{ {\left| R \right\rangle \equiv \left| 0 \right\rangle , \, \left| L \right\rangle \equiv \left| 1 \right\rangle } \right\}$ and the circularly polarized states ($\left| R \right\rangle$: right and $\left| L \right\rangle$: left) are related to the linearly polarized states ($\left| H \right\rangle$: horizontal and $\left| V \right\rangle$: vertical), using $\left| R \right\rangle \equiv \left( {\left| H \right\rangle + \left| V \right\rangle } \right)/\sqrt 2$ and $\left| L \right\rangle \equiv \left( {\left| H \right\rangle - \left| V \right\rangle } \right)/\sqrt 2$. For robustness against collective decoherence, we can encode arbitrary quantum information onto four-photon decoherence-free states, as follows:

3 $$\left| {\psi_{{{\text{PL}}}} } \right\rangle_{{{\text{ABCD}}}} = \, \alpha \left| {0_{{{\text{PL}}}} } \right\rangle_{{{\text{ABCD}}}} + \beta \left| {1_{{{\text{PL}}}} } \right\rangle_{{{\text{ABCD}}}} ,$$

where $\left| \alpha \right|^{2} + \left| \beta \right|^{2} = 1$. Through this encoding process (single logical qubit information onto a decoherence-free subspace), we can conserve the arbitrary quantum information encoded onto logical qubits under collective decoherence.

### Quantum dot confined in a single-sided optical cavity

In this section, we introduce the concept of a quantum dot (QD) within a cavity (QD-cavity system)^11,20,22,47,58,62–69^, which can induce the interaction of a photon and a singly charged QD (a negatively charged exciton:${\text{X}}^{ - }$). For the coherence of quantum systems in quantum information processing schemes, the systems of micropillar cavities have been widely utilized to construct quantum controlled gates^11,20,22,47,58,62–69^. Additionally, quantum information in the QD-cavity system can be effectively isolated from the environment for a long electron-spin coherence time (${\text{T}}_{{ 2}}^{{\text{ e}}}$ ~ ${\mu s}$)^70–75^ and a limited spin relaxation period (${\text{T}}_{{ 1}}^{{\text{ e}}}$ ~ ${\text{ms}}$)^76–79^.

**Figure 1:**
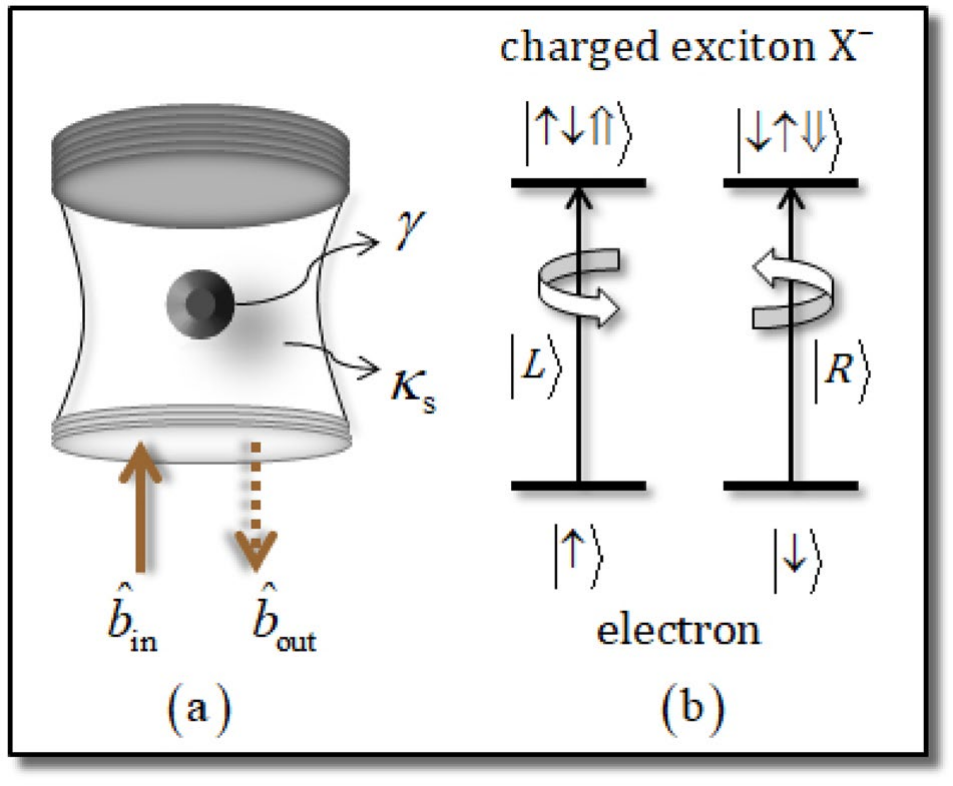
(**a**) Schematic of a singly charged QD inside a single-sided cavity, interacting with a photon (input and output field operators: $\hat{b}_{{{\text{in}}}}$ and $\hat{b}_{{{\text{out}}}}$), with a side-leakage rate ($\kappa_{s}$) of cavity mode and decay rate ($\gamma$) of ${\text{X}}^{ - }$. (**b**) By the spin selection rule in the QD, the induced interaction is $\left| \uparrow \right\rangle \to \left| { \uparrow \downarrow \Uparrow } \right\rangle$ ($\left| \downarrow \right\rangle \to \left| { \downarrow \uparrow \Downarrow } \right\rangle$), according to the photon polarization of $\left| L \right\rangle$ ($\left| R \right\rangle$).

In Fig. 1, the schematic of the QD-cavity system, Fig. 1a, and the spin selection rule, Fig. 1b, in the QD^11,20,22,47,58,62–69^ are presented with $\left| \uparrow \right\rangle \equiv \left| { + 1/2} \right\rangle {, }\left| \downarrow \right\rangle \equiv \left| { - 1/2} \right\rangle$ (the spin states of the excess electron), and $\left| \Uparrow \right\rangle \equiv \left| { + 3/2} \right\rangle , \, \left| \Downarrow \right\rangle \equiv \left| { - 3/2} \right\rangle$ (heavy-hole spin states). The single-sided cavity consists of two GaAs/Al(Ga)As distributed Bragg reflectors, DBR: the bottom DBR is partially reflective and the top DBR, 100% reflective, and a transverse index guide for the three-dimensional confinement of light. Figure 1a shows that a singly charged electron self-assembled QD is embedded in the center of the single-sided cavity. When an excess electron is injected into the QD, optical excitations can create a negatively charged exciton (${\text{X}}^{ - }$), as described in Fig. 1b. By the Pauli exclusion principle, if the spin state of the excess electron in the QD is in the state $\left| \uparrow \right\rangle$, then a polarization $\left| L \right\rangle$ of a photon can drive the state $\left| { \uparrow \downarrow \Uparrow } \right\rangle$ of ${\text{X}}^{ - }$. Moreover, if the spin state of the excess electron in the QD is $\left| \downarrow \right\rangle$ and the polarization of a photon is $\left| R \right\rangle$, through the interaction, the state $\left| { \downarrow \uparrow \Downarrow } \right\rangle$ of ${\text{X}}^{ - }$ can be created. The reflection coefficient $R\left( \omega \right)$, which is induced by the reflected photon from the interaction between the input photon pulse and the QD-cavity system, can be calculated by the Heisenberg equation of motion^57^ and the spin selection rule, with the ground state in the QD ($\left\langle {\hat{\sigma }_{Z} } \right\rangle = - 1$) for the steady state in the weak excitation approximation. Additionally, we can obtain the reflection coefficient, $R_{{\text{h}}}$ ($R_{{0}}$) of the hot (cold) cavity when the QD is coupled (uncoupled) to the cavity, depending on the spin selection rule of $\left| R \right\rangle \left| \downarrow \right\rangle , \, \left| L \right\rangle \left| \uparrow \right\rangle$ ($\left| R \right\rangle \left| \uparrow \right\rangle , \, \left| L \right\rangle \left| \downarrow \right\rangle$), with the coupling strength $g$ between ${\text{X}}^{ - }$ and the cavity mode, and the decay rate $\kappa$ of the cavity mode, as follows:

4 $$\begin{aligned} & \left[ {g \ne 0} \right]: \\\,&\quad R\left( \omega \right) = R_{{\text{h}}} \left( \omega \right) \equiv \left| {R_{{\text{h}}} \left( \omega \right)} \right|\exp \left[ {i\varphi_{{{\text{Rh}}}} \left( \omega \right)} \right] \, = \, 1 - \frac{{\kappa \left[ {i\left( {\omega_{{{\text{X}}^{ - } }} - \omega } \right) + \gamma /2} \right]}}{{\left[ {i\left( {\omega_{{{\text{X}}^{ - } }} - \omega } \right) + \gamma /2} \right]\left[ {i\left( {\omega_{c} - \omega } \right) + \kappa /2 + \kappa_{s} /2} \right] + g^{2} }}, \, \\ & \left[ {g = 0} \right]:\\\,&\quad R_{{0}} \left( \omega \right) \equiv \left| {R_{{0}} \left( \omega \right)} \right|\exp \left[ {i\varphi_{{{\text{R0}}}} \left( \omega \right)} \right] \, = \, 1 - \frac{\kappa }{{i\left( {\omega_{c} - \omega } \right) + \kappa /2 + \kappa_{s} /2}}, \\ \end{aligned}$$

where $\left| {R_{{\text{h}}} } \right|$ ($\left| {R_{{0}} } \right|$) and $\varphi_{{{\text{Rh}}}} = \arg [R_{{\text{h}}} ]$ ($\varphi_{{{\text{R0}}}} = \arg [R_{{0}} ]$) are the reflectance and phase shift of the hot (cold) cavity, respectively. $\omega_{{{\text{X}}^{ - } }}$, $\omega_{c}$, and $\omega$ are the frequencies of ${\text{X}}^{ - }$, the cavity mode, and the external field (photon), respectively. Thus, after the interaction between a photon and the QD-cavity system, the reflection operator $\hat{\text{R}}\left( \omega \right)$ from Eq. 4 is given by:

5 $${\hat{\text{R}}}\left( \omega \right) = \left| {R_{\text{h}} \left( \omega \right)} \right|e^{i{\varphi}_{\text{Rh}} \left( \omega \right)} \left( {\left| R \right\rangle \left\langle R \right| \otimes \left| \downarrow \right\rangle \left\langle \downarrow \right| + \left| L \right\rangle \left\langle L \right| \otimes \left| \uparrow \right\rangle \left\langle \uparrow \right|} \right) + \left| {R_{0} \left( \omega \right)} \right|e^{i{\varphi}_{{\text{R}}0} \left( \omega \right)} \left( {\left| R \right\rangle \left\langle R \right| \otimes \left| \uparrow \right\rangle \left\langle \uparrow \right| + \left| L \right\rangle \left\langle L \right| \otimes \left| \downarrow \right\rangle \left\langle \downarrow \right|} \right).$$

Here, if we take the experimental conditions of $\omega_{{{\text{X}}^{ - } }} = \omega_{c}$ (resonant interaction), such as a small side-leakage rate, $\kappa_{s} \ll \kappa$, a strong coupling strength, $g \gg \left( {\kappa , \, \gamma } \right)$, and a small $\gamma$ (~ several µeV)^80–82^, the reflection operators, $\hat{\text{R}}_{1}^{{{\text{Id}}}} \left( \omega \right)$ and $\hat{\text{R}}_{2}^{{{\text{Id}}}} \left( \omega \right)$, with regard to $\omega - \omega_{c}$, $2\left( {\omega - \omega_{c} } \right)/\kappa$: frequency detuning, in the ideal case (without vacuum noise and leaky modes, such as sideband leakage and absorption) can be calculated as:

6 $$\begin{aligned} & \left[ {\omega - \omega_{c} = \kappa /2} \right] \, \Rightarrow \\&\quad \hat{\text{R}}_{1}^{{{\text{Id}}}} \left( \omega \right) = \left( {\left| R \right\rangle \left\langle R \right| \otimes \left| \downarrow \right\rangle \left\langle \downarrow \right| + \left| L \right\rangle \left\langle L \right| \otimes \left| \uparrow \right\rangle \left\langle \uparrow \right|} \right) - i\left( {\left| R \right\rangle \left\langle R \right| \otimes \left| \uparrow \right\rangle \left\langle \uparrow \right| + \left| L \right\rangle \left\langle L \right| \otimes \left| \downarrow \right\rangle \left\langle \downarrow \right|} \right), \\ & \left[ {\omega - \omega_{c} = 0} \right] \, \Rightarrow \\&\quad \hat{\text{R}}_{2}^{{{\text{Id}}}} \left( \omega \right) = \left( {\left| R \right\rangle \left\langle R \right| \otimes \left| \downarrow \right\rangle \left\langle \downarrow \right| + \left| L \right\rangle \left\langle L \right| \otimes \left| \uparrow \right\rangle \left\langle \uparrow \right|} \right) - \left( {\left| R \right\rangle \left\langle R \right| \otimes \left| \uparrow \right\rangle \left\langle \uparrow \right| + \left| L \right\rangle \left\langle L \right| \otimes \left| \downarrow \right\rangle \left\langle \downarrow \right|} \right), \\ \end{aligned}$$

where the values of the reflectances and the phase shifts are $\left| {R_{{0}} } \right| = \left| {R_{{\text{h}}} } \right| \approx 1$ and $\varphi_{{{\text{Rh}}}} \approx 0 \, \left( { \approx 0} \right)$, $\varphi_{{{\text{R0}}}} \approx - \pi /2 \, \left( { \approx \pi } \right)$ from Eq. 4, according to the adjustment of the frequencies $\omega - \omega_{c} = \kappa /2 \, \left( { = 0} \right)$ between the external field and the cavity mode when $\kappa_{s}$ is negligible, with $g/\kappa = 2.4$ and $\gamma /\kappa = 0.1$^11,20,22,42,62,63,67^.

### Generation of four-photon decoherence-free states and the encoding process for single logical qubit information

**Figure 2:**
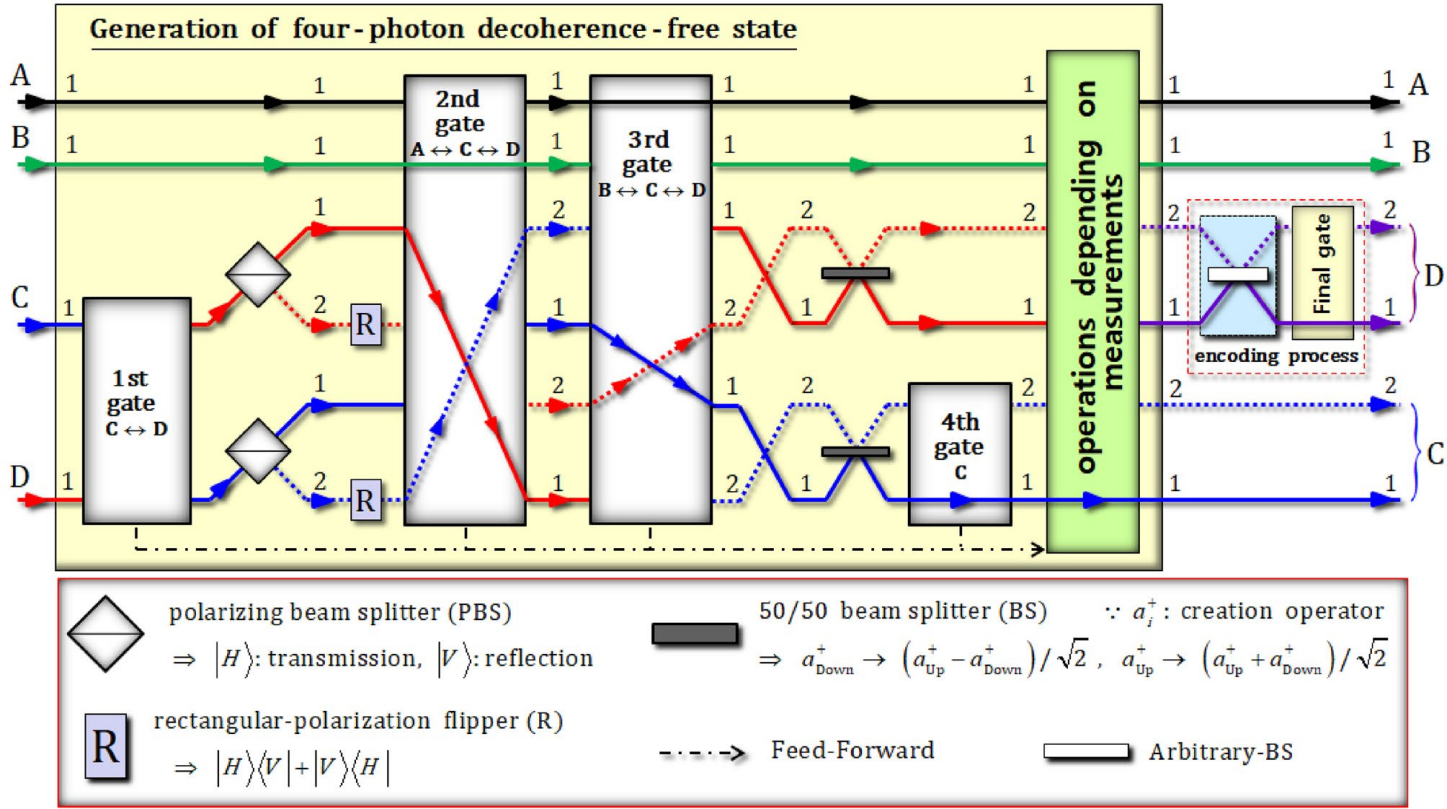
Encoding scheme for single logical qubit information onto four-photon decoherence-free states. This scheme is comprised of two parts: the generation of four-photon decoherence-free states and the encoding process. In the generation of the four-photon decoherence-free states, the four (1st, 2nd, 3rd, and 4th) gates employ the QD-cavity systems (QD1, 2, 3, and 4). The final gate in the encoding process also utilizes a QD-cavity system (QD5) to encode the single logical qubit information, with minimal collective decoherence.

In Fig. 2, we present the design of the scheme to encode single logical qubit information onto four-photon decoherence-free states using the QD-cavity systems and linearly optical devices. The scheme is composed of two parts: the generation of four-photon decoherence-free states and the process of encoding arbitrary quantum information. To obtain quantum information that is robust against collective decoherence^34,35,59^, our scheme can encode arbitrary quantum information onto four-photon decoherence-free states, (single logical qubit information), such as $\alpha \left| {0_{{\text{ PL}}} } \right\rangle_{{{\text{ABCD}}}} + \beta \left| {1_{{\text{ PL}}} } \right\rangle_{{{\text{ABCD}}}}$ in Eq. 3. To explain the process in detail, we first prepare the initial state as $\left| {\psi_{1} } \right\rangle_{{{\text{ABCD}}}} = \left| V \right\rangle_{{\text{A}}}^{1} \otimes \left| V \right\rangle_{{\text{B}}}^{1} \otimes \left( {\left| R \right\rangle_{{\text{C}}}^{1} + i\left| L \right\rangle_{{\text{C}}}^{1} } \right)/\sqrt 2 \otimes \left( {\left| R \right\rangle_{{\text{D}}}^{1} + i\left| L \right\rangle_{{\text{D}}}^{1} } \right)/\sqrt 2$ (product state of four photons). And, for the convenience, we define the expressions of path and photon, as $\left| {{\text{state}}} \right\rangle_{{{\text{photon}}}}^{{{\text{path}}}}$.

#### 1st gate [(photons C, D) ↔ QD1]

**Figure 3:**
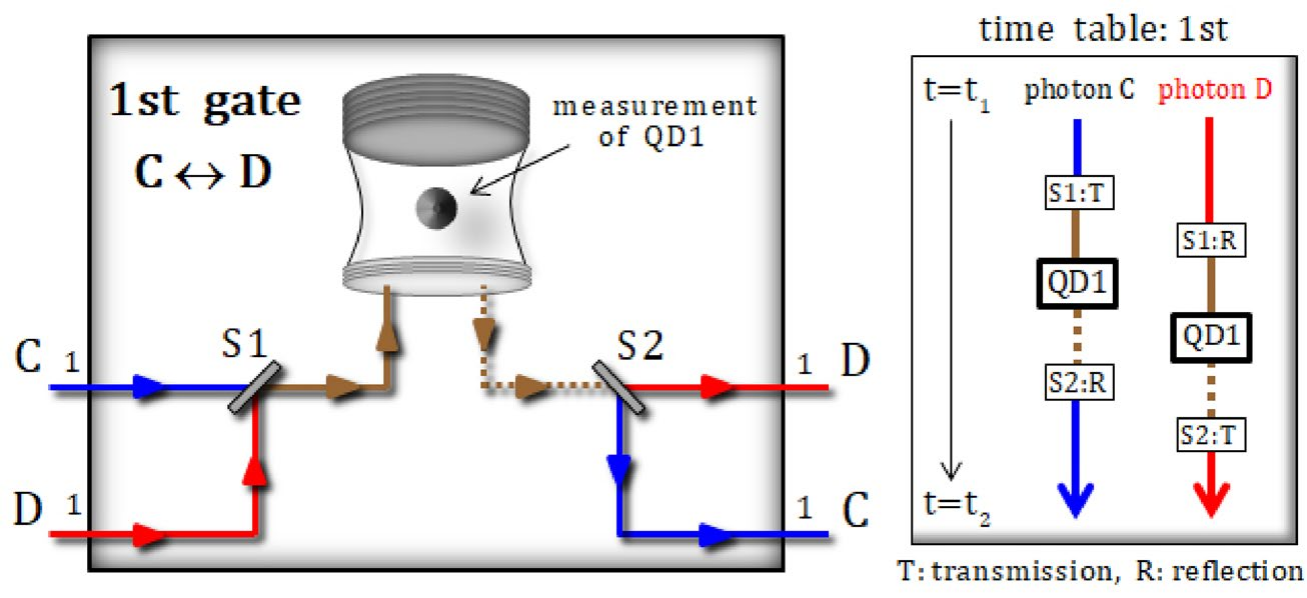
Details of the first gate (QD1) in Fig. 2. The sequential interactions between two photons (C and D) and an electron spin 1 in QD1 utilize the reflection operator ${\hat{\text{R}}}_{1}^{{{\text{Id}}}} \left( \omega \right)$ in Eq. 6 with a frequency of $\omega - \omega_{c} = \kappa /2$. The prepared excess electron spin state is in the state $\left| { +_{{\text{e}}} } \right\rangle_{1}$. This electron spin 1 is then measured after the interactions, in accordance with time table for switches (S1 and S2).

In the first gate (Fig. 3), two photons (C and D) and an electron spin 1 $\left| { +_{{\text{e}}} } \right\rangle_{{1}}$ (the prepared electron 1) in QD1, sequentially interact with each other, according to the reflection operator $\hat{\text{R}}_{1}^{{{\text{Id}}}} \left( \omega \right)$ in Eq. 6 with a frequency detuning of $\omega - \omega_{c} = \kappa /2$, where the spin states are defined as $\left| { \pm_{{\text{e}}} } \right\rangle = \left( {\left| \uparrow \right\rangle \pm \left| \downarrow \right\rangle } \right)/\sqrt 2$.

After the operation of the first gate, which interacts with two photons (C and D) and QD1 in sequence, according to time table (${\text{t}}_{{1}} \to {\text{t}}_{{2}}$), the state of pre-measurement is given by:

7 $$\begin{aligned} & \left| {\psi_{{1}} } \right\rangle_{{{\text{ABCD}}}} \otimes \left| { +_{{\text{e}}} } \right\rangle_{1} {\mathop{\longrightarrow}\limits^{{1{\text{st}}\,{\text{gate}}}}} \, \left| {\psi_{{2}}^{{\text{i}}} } \right\rangle_{{{\text{1ABCD}}}} \\ &\quad= \frac{1}{\sqrt 2 }\left\{ {\left| { +_{{\text{e}}} } \right\rangle_{1} \otimes \left| {VV} \right\rangle_{{{\text{AB}}}}^{{{11}}} \otimes \frac{1}{\sqrt 2 }\left( {\left| {HH} \right\rangle - \left| {VV} \right\rangle } \right)_{{{\text{CD}}}}^{{{11}}} - \left| { -_{{\text{e}}} } \right\rangle_{1} \otimes \left| {VV} \right\rangle_{{{\text{AB}}}}^{{{11}}} \otimes \frac{1}{\sqrt 2 }\left( {\left| {HH} \right\rangle + \left| {VV} \right\rangle } \right)_{{{\text{CD}}}}^{{{11}}} } \right\}, \end{aligned}$$

where the interactions of the QD-cavity system are expressed as the reflection operator $\hat{\text{R}}_{1}^{{{\text{Id}}}} \left( \omega \right)$ in Eq. 6, with a frequency of $\omega - \omega_{c} = \kappa /2$ between the external field and the cavity mode. For example, if we assume that the result of a measurement in QD1 is in the state $\left| { -_{{\text{e}}} } \right\rangle_{1}$, the post-measurement state $\left| {\psi_{{2}}^{{\text{f}}} } \right\rangle_{{{\text{ABCD}}}}$ will be:

8 $$\left| {\psi_{{2}}^{{\text{i}}} } \right\rangle_{{{\text{1ABCD}}}} {\mathop{\longrightarrow}\limits^{{{\text{measurement}}}}} \, \left[ {{\text{result: }}\left| { -_{{\text{e}}} } \right\rangle_{{1}} } \right] \, \to \, \left| {\psi_{{2}}^{{\text{f}}} } \right\rangle_{{{\text{ABCD}}}} = \left| {VV} \right\rangle_{{{\text{AB}}}}^{{{11}}} \otimes \frac{1}{\sqrt 2 }\left( {\left| {HH} \right\rangle + \left| {VV} \right\rangle } \right)_{{{\text{CD}}}}^{{{11}}} .$$

Subsequently, two polarizing beam splitters (PBSs) and rectangular-polarization flippers (Rs), as described in Fig. 2, operate to affect the state $\left| {\psi_{{2}}^{{\text{f}}} } \right\rangle_{{{\text{ABCD}}}}$ of the first gate, as follows:

9 $$\left| {\psi_{{2}}^{{\text{f}}} } \right\rangle_{{{\text{ABCD}}}} {\mathop{\longrightarrow}\limits^{{\text{PBSs, Rs}}}} \, \left| {\psi_{3} } \right\rangle_{{{\text{ABCD}}}} = \left| {VV} \right\rangle_{{{\text{AB}}}}^{{{11}}} \otimes \frac{1}{\sqrt 2 }\left( {\left| {HH} \right\rangle_{{{\text{CD}}}}^{{{11}}} + \left| {HH} \right\rangle_{{{\text{CD}}}}^{{{22}}} } \right).$$

#### 2nd gate [(photons A, C, D) ↔ QD2] and 3rd gate [(photons B, C, D) ↔ QD3]

**Figure 4:**
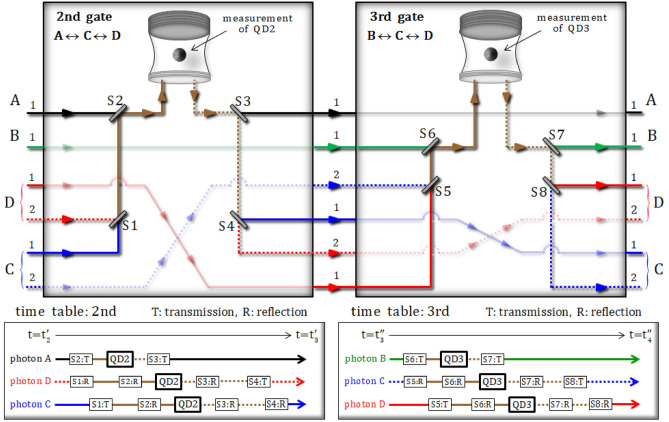
The second and third gate (QD2 and QD3) in Fig. 2. The sequential interactions between three photons, A, C, and D (B, C, and D), and an electron spin 2 (3) in QD2 (QD3) are operated by the reflection operator ${\hat{\text{R}}}_{1}^{{{\text{Id}}}} \left( \omega \right)$ in Eq. 6, with a frequency of $\omega - \omega_{c} = \kappa /2$. The prepared excess electron spin state in QD2 [QD3] is in the state $\left| { +_{{\text{e}}} } \right\rangle_{2}$ ($\left| { +_{{\text{e}}} } \right\rangle_{3}$). This electron spin 1 (2) is then measured after the interactions, in accordance with the second [third] time table for switches, S1, S2, S3, and S4 (S5, S6, S7, and S8), in sequence.

In the second (third) gate, depicted in Fig. 4, three photons, A, C, and D (B, C, and D), and an electron spin 2 (3), [$\left| { +_{{\text{e}}} } \right\rangle_{{2}}$ ($\left| { +_{{\text{e}}} } \right\rangle_{{3}}$): the prepared electron 2 (3)] in QD2 (QD3) sequentially interact with each other, according to the reflection operator $\hat{\text{R}}_{1}^{{{\text{Id}}}} \left( \omega \right)$ in Eq. 6, with a frequency detuning of $\omega - \omega_{c} = \kappa /2$. After the operation of the second gate, which interacts with three photons (A, C, and D) and QD2 in sequence, according to time table (${\text{t}}_{2}^{\prime } \to {\text{t}}_{3}^{\prime }$), the state of the pre-measurement is given by:

10 $$\begin{aligned} & \left| {\psi_{3} } \right\rangle_{{{\text{ABCD}}}} \otimes \left| { +_{{\text{e}}} } \right\rangle_{2} {\mathop{\longrightarrow}\limits^{{2{\text{nd}}\;{\text{gate}}}}} \, \left| {\psi_{{4}}^{{\text{i}}} } \right\rangle_{{{\text{2BACD}}}} \\&\quad = \frac{ - i}{{\sqrt 2 }}\left| { +_{{\text{e}}} } \right\rangle_{2} \otimes \left| V \right\rangle_{{\text{B}}}^{{1}} \otimes \frac{1}{\sqrt 2 }\left\{ {\frac{1}{\sqrt 2 }\left( {\left| {RL} \right\rangle - \left| {LR} \right\rangle } \right)_{{{\text{AC}}}}^{{{11}}} \otimes \left| H \right\rangle_{{\text{D}}}^{{1}}+ \frac{1}{\sqrt 2 }\left( {\left| {RL} \right\rangle - \left| {LR} \right\rangle } \right)_{{{\text{AD}}}}^{{{12}}} \otimes \left| H \right\rangle_{{\text{C}}}^{{2}} } \right\} \\ &\quad\quad+ \frac{ - 1}{{\sqrt 2 }}\left| { -_{{\text{e}}} } \right\rangle_{2} \otimes \left| V \right\rangle_{{\text{B}}}^{{1}} \otimes \frac{1}{\sqrt 2 }\left\{ {\frac{1}{\sqrt 2 }\left( {\left| {RR} \right\rangle + \left| {LL} \right\rangle } \right)_{{{\text{AC}}}}^{{{11}}} \otimes \left| H \right\rangle_{{\text{D}}}^{{1}} + \frac{1}{\sqrt 2 }\left( {\left| {RR} \right\rangle + \left| {LL} \right\rangle } \right)_{{{\text{AD}}}}^{{{12}}} \otimes \left| H \right\rangle_{{\text{C}}}^{{2}} } \right\}, \\ \end{aligned}$$

where the reflection operator $\hat{\text{R}}_{1}^{{{\text{Id}}}} \left( \omega \right)$ is given by Eq. 6, with $\omega - \omega_{c} = \kappa /2$. If the measurement outcome of QD2 is in the state $\left| { +_{{\text{e}}} } \right\rangle_{2}$, the post-measurement state $\left| {\psi_{{4}}^{{\text{f}}} } \right\rangle_{{{\text{BACD}}}}$ is then given by:

11 $$\begin{aligned} & \left| {\psi_{{4}}^{{\text{i}}} } \right\rangle_{{{\text{2BACD}}}} {\mathop{\longrightarrow}\limits^{{{\text{measurement}}}}} \, \left[ {{\text{result: }}\left| { +_{{\text{e}}} } \right\rangle_{{2}} } \right] \, \to \, \left| {\psi_{{4}}^{{\text{f}}} } \right\rangle_{{{\text{BACD}}}} \\ &\quad= \left| V \right\rangle_{{\text{B}}}^{{1}} \otimes \frac{1}{\sqrt 2 }\left\{ {\frac{1}{\sqrt 2 }\left( {\left| {RL} \right\rangle - \left| {LR} \right\rangle } \right)_{{{\text{AC}}}}^{{{11}}} \otimes \left| H \right\rangle_{{\text{D}}}^{{1}} + \frac{1}{\sqrt 2 }\left( {\left| {RL} \right\rangle - \left| {LR} \right\rangle } \right)_{{{\text{AD}}}}^{{{12}}} \otimes \left| H \right\rangle_{{\text{C}}}^{{2}} } \right\}. \end{aligned}$$

In the third gate, three photons (B, C, and D) of the state $\left| {\psi_{{4}}^{{\text{f}}} } \right\rangle_{{{\text{BACD}}}}$ interact with an electron spin state $\left| { +_{{\text{e}}} } \right\rangle_{{3}}$ in QD3, as follows:

12 $$\begin{aligned} & \left| {\psi_{{4}}^{{\text{f}}} } \right\rangle_{{{\text{BACD}}}} \otimes \left| { +_{{\text{e}}} } \right\rangle_{3} {\mathop{\longrightarrow}\limits^{{\text{3rd gate}}}} \, \left| {\psi_{{5}}^{{\text{i}}} } \right\rangle_{{{\text{3ABCD}}}} \\&\quad = \frac{ - i}{{\sqrt 2 }}\left| { +_{{\text{e}}} } \right\rangle_{3} \otimes \frac{1}{\sqrt 2 }\left\{ {\frac{1}{\sqrt 2 }\left( {\left| {RL} \right\rangle - \left| {LR} \right\rangle } \right)_{{{\text{AC}}}}^{{{11}}} \otimes \frac{1}{\sqrt 2 }\left( {\left| {RL} \right\rangle - \left| {LR} \right\rangle } \right)_{{{\text{BD}}}}^{{{11}}} + \frac{1}{\sqrt 2 }\left( {\left| {RL} \right\rangle - \left| {LR} \right\rangle } \right)_{{{\text{AD}}}}^{{{12}}} \otimes \frac{1}{\sqrt 2 }\left( {\left| {RL} \right\rangle - \left| {LR} \right\rangle } \right)_{{{\text{BC}}}}^{{{12}}} } \right\} \\ &\qquad + \frac{ - 1}{{\sqrt 2 }}\left| { -_{{\text{e}}} } \right\rangle_{3} \otimes \frac{1}{\sqrt 2 }\left\{ {\frac{1}{\sqrt 2 }\left( {\left| {RL} \right\rangle - \left| {LR} \right\rangle } \right)_{{{\text{AC}}}}^{{{11}}} \otimes \frac{1}{\sqrt 2 }\left( {\left| {RR} \right\rangle + \left| {LL} \right\rangle } \right)_{{{\text{BD}}}}^{{{11}}} + \frac{1}{\sqrt 2 }\left( {\left| {RL} \right\rangle - \left| {LR} \right\rangle } \right)_{{{\text{AD}}}}^{{{12}}} \otimes \frac{1}{\sqrt 2 }\left( {\left| {RR} \right\rangle + \left| {LL} \right\rangle } \right)_{{{\text{BC}}}}^{{{12}}} } \right\}, \\ \end{aligned}$$

where the reflection operator $\hat{\text{R}}_{1}^{{{\text{Id}}}} \left( \omega \right)$ is given by Eq. 6, with $\omega - \omega_{c} = \kappa /2$, according to time table (${\text{t}}_{3}^{\prime \prime } \to {\text{t}}_{4}^{\prime \prime }$). For a measurement outcome in the state $\left| { +_{{\text{e}}} } \right\rangle_{{3}}$ of QD3, we obtain the output state $\left| {\psi_{{5}}^{{\text{f}}} } \right\rangle_{{{\text{ABCD}}}}$ from the third gate, as follows:

13 $$\begin{aligned} &\left| {\psi_{{5}}^{{\text{i}}} } \right\rangle_{{{\text{3ABCD}}}} {\mathop{\longrightarrow}\limits^{{{\text{measurement}}}}} \, \left[ {{\text{result: }}\left| { +_{{\text{e}}} } \right\rangle_{{3}} } \right] \,\to  \, \left| {\psi_{5}^{{\text{f}}} } \right\rangle_{{{\text{ABCD}}}}\\ &\qquad = \frac{1}{\sqrt 2 }\left\{ {\frac{1}{\sqrt 2 }\left( {\left| {RL} \right\rangle - \left| {LR} \right\rangle } \right)_{{{\text{AC}}}}^{{{11}}} \otimes \frac{1}{\sqrt 2 }\left( {\left| {RL} \right\rangle - \left| {LR} \right\rangle } \right)_{{{\text{BD}}}}^{{{11}}} + \frac{1}{\sqrt 2 }\left( {\left| {RL} \right\rangle - \left| {LR} \right\rangle } \right)_{{{\text{AD}}}}^{{{12}}} \otimes \frac{1}{\sqrt 2 }\left( {\left| {RL} \right\rangle - \left| {LR} \right\rangle } \right)_{{{\text{BC}}}}^{{{12}}} } \right\}. \\ \end{aligned}$$

Subsequently, as described in Fig. 2, two 50:50 beam splitters (BSs) are applied to two photons, C and D, of the output state $\left| {\psi_{{5}}^{{\text{f}}} } \right\rangle_{{{\text{ABCD}}}}$, as follows:

14 $$\begin{aligned} & \left| {\psi_{{5}}^{{\text{f}}} } \right\rangle_{{{\text{ABCD}}}} {\mathop{\longrightarrow}\limits^{{{\text{BSs}}}}} \, \left| {\psi_{6} } \right\rangle_{{{\text{ABCD}}}} \\&\quad = \frac{1}{\sqrt 2 }\left[ {\frac{1}{2}\left\{ {\frac{1}{2}\left( { - \left| {RLRL} \right\rangle + \left| {RLLR} \right\rangle + \left| {LRRL} \right\rangle - \left| {LRLR} \right\rangle } \right)_{{{\text{ABCD}}}}^{{{1112}}} } \right\}} \right.\\&\qquad + \left. { \frac{\sqrt 3 }{2}\left\{ {\frac{1}{{\sqrt {12} }}\left( {2\left| {RRLL} \right\rangle + 2\left| {LLRR} \right\rangle - \left| {RLRL} \right\rangle - \left| {RLLR} \right\rangle - \left| {LRRL} \right\rangle - \left| {LRLR} \right\rangle } \right)_{{{\text{ABCD}}}}^{{{1111}}} } \right\}} \right] \\ &\quad\quad \left[ { + \frac{1}{\sqrt 2 }\frac{1}{2}\left\{ {\frac{1}{2}\left( { - \left| {RLRL} \right\rangle + \left| {RLLR} \right\rangle + \left| {LRRL} \right\rangle - \left| {LRLR} \right\rangle } \right)_{{{\text{ABCD}}}}^{{{1121}}} } \right\} } \right.\\&\qquad + \left. { \frac{\sqrt 3 }{2}\left\{ {\frac{1}{{\sqrt {12} }}\left( {2\left| {RRLL} \right\rangle + 2\left| {LLRR} \right\rangle - \left| {RLRL} \right\rangle - \left| {RLLR} \right\rangle - \left| {LRRL} \right\rangle - \left| {LRLR} \right\rangle } \right)_{{{\text{ABCD}}}}^{{{1122}}} } \right\}} \right] \\ \end{aligned}$$

#### 4th gate [photon C ↔ QD4] and the operations dependent on the measurements

**Figure 5:**
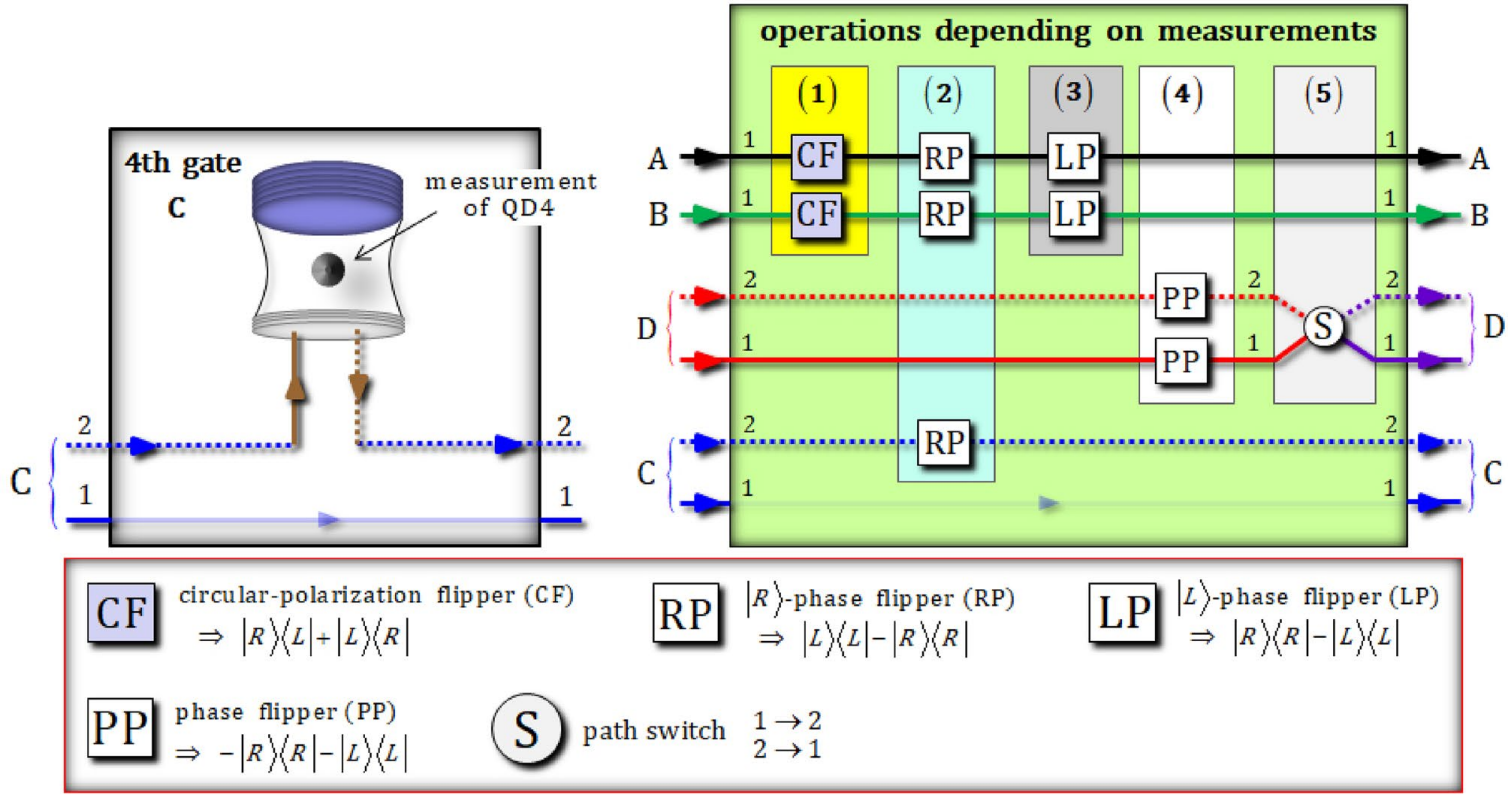
The fourth gate (QD4) and the operations dependent on the measurements in Fig. 2. For the alignment of the path of photon C, the fourth gate utilizes the reflection operator ${\hat{\text{R}}}_{2}^{{{\text{Id}}}} \left( \omega \right)$ given by Eq. 6 with a frequency of $\omega - \omega_{c} = 0$ for the interaction between a photon C and an electron spin 4, which is prepared to the state $\left| { +_{{\text{e}}} } \right\rangle_{4}$, in QD4. Subsequently, due to the measurement outcomes of QD1, QD2, QD3, and QD4, the operations, depending on measurements, are performed on photons A, B, C, and D by Feed-Forward.

In the fourth gate (Fig. 5), the reflection operator $\hat{\text{R}}_{2}^{{{\text{Id}}}} \left( \omega \right)$, which is given by Eq. 6, with $\omega - \omega_{c} = 0$, performs an operation between a photon C and an electron spin 4 ($\left| { +_{{\text{e}}} } \right\rangle_{{4}}$: the prepared electron 4) in QD4. Subsequently, in the operations depending on measurements, diverse operators [circular-polarization flippers (CFs), $\left| R \right\rangle$- and $\left| L \right\rangle$-phase flippers (RPs and LPs), phase flippers (PPs), and a path switch] are applied to the output state from the fourth gate, according to the measurement outcomes of QD1, QD2, QD3, and QD4. After the interaction in the fourth gate, between photon C and QD4 of the state $\left| {\psi_{6} } \right\rangle_{{{\text{ABCD}}}}$, the state of pre-measurement $\left| {\psi_{7}^{{\text{i}}} } \right\rangle_{{{\text{ABCD}}}}$ is given by:

15 $$\begin{aligned} & \left| {\psi_{6} } \right\rangle_{{{\text{ABCD}}}} \otimes \left| { +_{{\text{e}}} } \right\rangle_{4} {\mathop{\longrightarrow}\limits^{{\text{4th gate}}}} \, \left| {\psi_{{7}}^{{\text{i}}} } \right\rangle_{{{\text{4ABCD}}}} \\ &\quad = \frac{1}{\sqrt 2 }\left| { +_{{\text{e}}} } \right\rangle_{4} \otimes \left[ {\frac{1}{2}\left\{ {\frac{1}{2}\left( { - \left| {RLRL} \right\rangle + \left| {RLLR} \right\rangle + \left| {LRRL} \right\rangle - \left| {LRLR} \right\rangle } \right)_{{{\text{ABCD}}}}^{{{1112}}} } \right\}}\right. \\&\qquad \left.{+ \frac{\sqrt 3 }{2}\left\{ {\frac{1}{{\sqrt {12} }}\left( {2\left| {RRLL} \right\rangle + 2\left| {LLRR} \right\rangle - \left| {RLRL} \right\rangle - \left| {RLLR} \right\rangle - \left| {LRRL} \right\rangle - \left| {LRLR} \right\rangle } \right)_{{{\text{ABCD}}}}^{{{1111}}} } \right\}} \right] \\ &\quad\quad + \frac{1}{\sqrt 2 }\left| { -_{{\text{e}}} } \right\rangle_{4} \otimes \left[ {\frac{1}{2}\left\{ {\frac{1}{2}\left( {\left| {RLRL} \right\rangle + \left| {RLLR} \right\rangle - \left| {LRRL} \right\rangle - \left| {LRLR} \right\rangle } \right)_{{{\text{ABCD}}}}^{{{1121}}} } \right\} }\right. \\&\qquad \left.{+ \frac{\sqrt 3 }{2}\left\{ {\frac{1}{{\sqrt {12} }}\left( {2\left| {RRLL} \right\rangle - 2\left| {LLRR} \right\rangle + \left| {RLRL} \right\rangle - \left| {RLLR} \right\rangle + \left| {LRRL} \right\rangle - \left| {LRLR} \right\rangle } \right)_{{{\text{ABCD}}}}^{{{1122}}} } \right\}} \right] \\ \end{aligned}$$

where the reflection operator $\hat{\text{R}}_{2}^{{{\text{Id}}}} \left( \omega \right)$ is given by Eq. 6, with $\omega - \omega_{c} = 0$.

Subsequently, if we assume that the result of a measurement in QD4 is in the state $\left| { +_{{\text{e}}} } \right\rangle_{4}$, the post-measurement state $\left| {\psi_{{7}}^{{\text{f}}} } \right\rangle_{{{\text{ABCD}}}}$ will be:

16 $$\begin{aligned} & \left| {\psi_{{7}}^{{\text{i}}} } \right\rangle_{{{\text{4ABCD}}}} {\mathop{\longrightarrow}\limits^{{{\text{measurement}}}}} \, \left[ {{\text{result: }}\left| { +_{{\text{e}}} } \right\rangle_{{4}} } \right] \, \to \, \left| {\psi_{{7}}^{{\text{f}}} } \right\rangle_{{{\text{ABCD}}}} \\&\quad = \frac{1}{2}\left\{ {\frac{1}{2}\left( { - \left| {RLRL} \right\rangle + \left| {RLLR} \right\rangle + \left| {LRRL} \right\rangle - \left| {LRLR} \right\rangle } \right)_{{{\text{ABCD}}}}^{{{1112}}} } \right\} \\ &\quad\quad+ \frac{\sqrt 3 }{2}\left\{ {\frac{1}{{\sqrt {12} }}\left( {2\left| {RRLL} \right\rangle + 2\left| {LLRR} \right\rangle - \left| {RLRL} \right\rangle - \left| {RLLR} \right\rangle - \left| {LRRL} \right\rangle - \left| {LRLR} \right\rangle } \right)_{{{\text{ABCD}}}}^{{{1111}}} } \right\}. \\ \end{aligned}$$

For the encoding of (arbitrary) quantum information onto four-photon decoherence-free states, (single logical qubit information), we require the superposed state of the four-photon decoherence-free states, $\left\{ {\left| {0_{{\text{ PL}}} } \right\rangle , \, \left| {1_{{\text{ PL}}} } \right\rangle } \right\}{: } \approx \left| {0_{{\text{ PL}}} } \right\rangle_{{{\text{ABCD}}}} + \left| {1_{{\text{ PL}}} } \right\rangle_{{{\text{ABCD}}}}$ in Eq. 2 (the superposition of logical qubits). Thus, our scheme utilizes the operations, depending on measurements, using Feed-Forward, as described in Fig. 5, to transform a superposed state four-photon decoherence-free subspace according to the measurement outcomes of four electron spin states in the QD-cavity systems (QD1 ~ QD4). In Table 1, all possible operations, due to the measurement outcomes of electrons spins 1 ~ 4 in QD1 ~ QD4 (polarization flippers, phase flippers, and path switch), are summarized, to apply to the output state $\left| {\psi_{{7}}^{{\text{f}}} } \right\rangle_{{{\text{ABCD}}}}$ of the fourth gate by Feed-Forward for the generation of a superposition state of four-photon decoherence-free states (logical qubits: $\left| {0_{{\text{ PL}}} } \right\rangle$ and $\left| {1_{{\text{ PL}}} } \right\rangle$). Till this point, we have assumed the measurement outcomes of each of the electron spin states in QD1 ~ QD4. From the results of Eqs. 8, 11, 13, and 16, the measurement outcome of QD1 ~ QD4 is in the state $\left| { -_{{\text{e}}} } \right\rangle_{1} \left| { +_{{\text{e}}} } \right\rangle_{2} \left| { +_{{\text{e}}} } \right\rangle_{3} \left| { +_{{\text{e}}} } \right\rangle_{4}$. We can subsequently apply a RP ($\left| R \right\rangle$-phase flipper from part 4 of the operations dependent on measurements) to path 2 of photon D, as (D-2), in the state $\left| {\psi_{{7}}^{{\text{f}}} } \right\rangle_{{{\text{ABCD}}}}$ by Feed-Forward.

**Table 1:** For a single type of the superposed four-photon decoherence-free states, the operations (circular-polarization flippers (CFs), $\left| R \right\rangle$- and $\left| L \right\rangle$-phase flippers (RPs and LPs), phase flippers (PPs), and a path switch] by Feed-Forward in parts (1), (2), (3), (4), and (5) should be applied to the output state of the fourth gate, due to the measurement results of the QDs. Here, we assign “O” and “N” to mean “Operation” and “No operation” of the Feed-Forward.

Results of QDs (1, 2, 3, 4)		Operations dependent on results ∵(photon-path)					Result state:$\left| {\psi_{8} } \right\rangle_{{{\text{ABCD}}}}$
		(1)	(2)	(3)	(4)	(5)	
$\left| { +_{{\text{e}}} } \right\rangle_{1} \left| { +_{{\text{e}}} } \right\rangle_{2} \left| { +_{{\text{e}}} } \right\rangle_{3}$	$\left| { +_{{\text{e}}} } \right\rangle_{4}$	N	N	N	(D-1)	O	$\frac{1}{2}\left| {0_{{\text{ PL}}} } \right\rangle_{{{\text{ABCD}}}}^{1112} + \frac{\sqrt 3 }{2}\left| {1_{{\text{ PL}}} } \right\rangle_{{{\text{ABCD}}}}^{1111}$
$\left| { -_{{\text{e}}} } \right\rangle_{1} \left| { +_{{\text{e}}} } \right\rangle_{2} \left| { +_{{\text{e}}} } \right\rangle_{3}$		N	N	N	(D-2)	N	
$\left| { +_{{\text{e}}} } \right\rangle_{1} \left| { -_{{\text{e}}} } \right\rangle_{2} \left| { -_{{\text{e}}} } \right\rangle_{3}$		(A),(B)	(A),(B)	N	(D-1)	O	
$\left| { -_{{\text{e}}} } \right\rangle_{1} \left| { -_{{\text{e}}} } \right\rangle_{2} \left| { -_{{\text{e}}} } \right\rangle_{3}$		(A),(B)	(A),(B)	N	(D-2)	N	
$\left| { +_{{\text{e}}} } \right\rangle_{1} \left| { -_{{\text{e}}} } \right\rangle_{2} \left| { +_{{\text{e}}} } \right\rangle_{3}$		(A)	N	(A)	(D-1)	O	
$\left| { -_{{\text{e}}} } \right\rangle_{1} \left| { -_{{\text{e}}} } \right\rangle_{2} \left| { +_{{\text{e}}} } \right\rangle_{3}$		(A)	N	(A)	(D-2)	N	
$\left| { +_{{\text{e}}} } \right\rangle_{1} \left| { +_{{\text{e}}} } \right\rangle_{2} \left| { -_{{\text{e}}} } \right\rangle_{3}$		(B)	N	(B)	(D-1)	O	
$\left| { -_{{\text{e}}} } \right\rangle_{1} \left| { +_{{\text{e}}} } \right\rangle_{2} \left| { -_{{\text{e}}} } \right\rangle_{3}$		(B)	N	(B)	(D-2)	N	
$\left| { +_{{\text{e}}} } \right\rangle_{1} \left| { +_{{\text{e}}} } \right\rangle_{2} \left| { +_{{\text{e}}} } \right\rangle_{3}$	$\left| { -_{{\text{e}}} } \right\rangle_{4}$	N	(C)	N	(D-2)	N	$\frac{1}{2}\left| {0_{{\text{ PL}}} } \right\rangle_{{{\text{ABCD}}}}^{1122} + \frac{\sqrt 3 }{2}\left| {1_{{\text{ PL}}} } \right\rangle_{{{\text{ABCD}}}}^{1121}$
$\left| { -_{{\text{e}}} } \right\rangle_{1} \left| { +_{{\text{e}}} } \right\rangle_{2} \left| { +_{{\text{e}}} } \right\rangle_{3}$		N	(C)	N	(D-1)	O	
$\left| { +_{{\text{e}}} } \right\rangle_{1} \left| { -_{{\text{e}}} } \right\rangle_{2} \left| { -_{{\text{e}}} } \right\rangle_{3}$		(A),(B)	(A),(B),(C)	N	(D-2)	N	
$\left| { -_{{\text{e}}} } \right\rangle_{1} \left| { -_{{\text{e}}} } \right\rangle_{2} \left| { -_{{\text{e}}} } \right\rangle_{3}$		(A),(B)	(A),(B),(C)	N	(D-1)	O	
$\left| { +_{{\text{e}}} } \right\rangle_{1} \left| { -_{{\text{e}}} } \right\rangle_{2} \left| { +_{{\text{e}}} } \right\rangle_{3}$		(A)	(C)	(A)	(D-2)	N	
$\left| { -_{{\text{e}}} } \right\rangle_{1} \left| { -_{{\text{e}}} } \right\rangle_{2} \left| { +_{{\text{e}}} } \right\rangle_{3}$		(A)	(C)	(A)	(D-1)	O	
$\left| { +_{{\text{e}}} } \right\rangle_{1} \left| { +_{{\text{e}}} } \right\rangle_{2} \left| { -_{{\text{e}}} } \right\rangle_{3}$		(B)	(C)	(B)	(D-2)	N	
$\left| { -_{{\text{e}}} } \right\rangle_{1} \left| { +_{{\text{e}}} } \right\rangle_{2} \left| { -_{{\text{e}}} } \right\rangle_{3}$		(B)	(C)	(B)	(D-1)	O	

Finally, as listed in Table 1, we can obtain the output state $\left| {\psi_{8} } \right\rangle_{{{\text{ABCD}}}}$ (the superposition of logical qubits) from the generation of decoherence-free states, as follows:

17 $$\left| {\psi_{7}^{{\text{f}}} } \right\rangle_{{{\text{ABCD}}}} {\mathop{\longrightarrow}\limits^{{\text{Feed }{-}\text{ Forward}}}} \, \left| {\psi_{8} } \right\rangle_{{{\text{ABCD}}}} = \frac{1}{2}\left| {0_{{\text{ PL}}} } \right\rangle_{{{\text{ABCD}}}}^{1112} + \frac{\sqrt 3 }{2}\left| {1_{{\text{ PL}}} } \right\rangle_{{{\text{ABCD}}}}^{1111} ,$$

where the four-photon decoherence-free states ($\left| {0_{{\text{ PL}}} } \right\rangle$ and $\left| {1_{{\text{ PL}}} } \right\rangle$) are given in Eq. 2. Furthermore, other types of superposed states from the fourth gate can also be transformed to the superposed state of the four-photon decoherence-free states $\left\{ {\left| {0_{{\text{ PL}}} } \right\rangle , \, \left| {1_{{\text{ PL}}} } \right\rangle } \right\}$ in Eq. 2 (the superposition of logical qubits) by the operation of Feed-Forward in Table 1.

#### Encoding process for single logical qubit information

**Figure 6:**
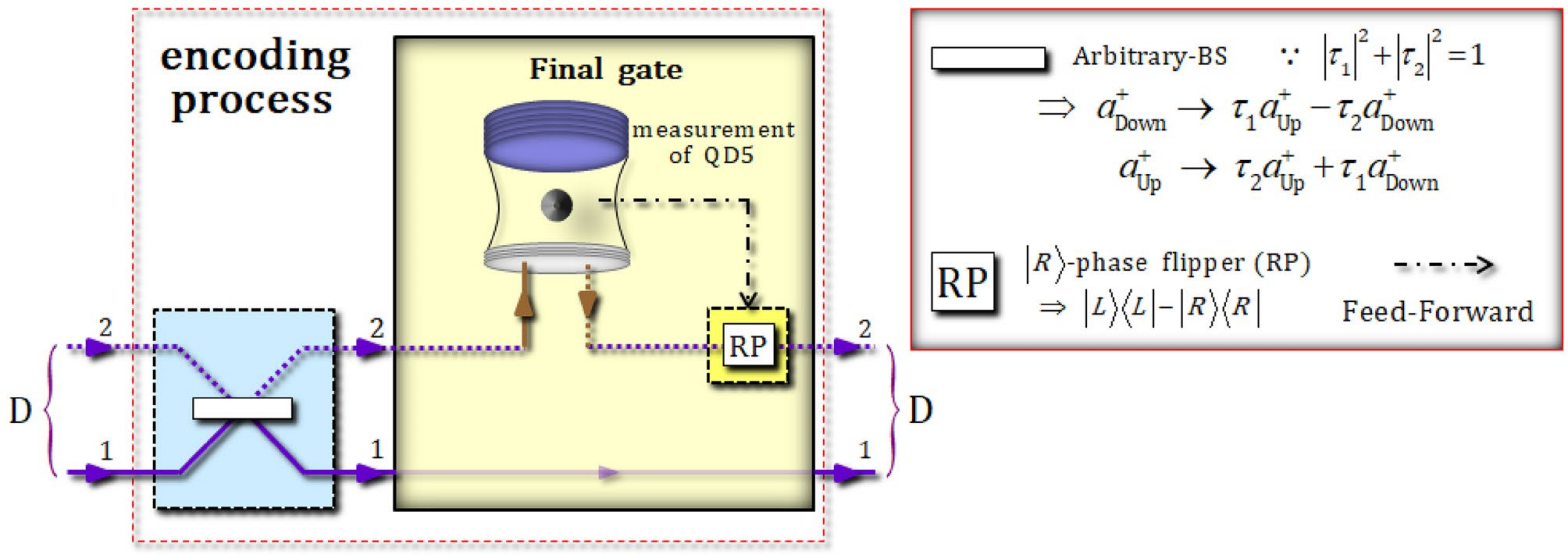
The encoding process (an arbitrary-BS and the final gate) in Fig. 2. To encode the arbitrary quantum information onto four-photon decoherence-free states, (single logical qubit information), the arbitrary-BS, having a transmission ($\tau_{1}$) and reflection ($\tau_{2}$) of arbitrary probabilities, is applied to photon D. The final gate then interacts with photon D using QD5, by the reflection operator ${\hat{\text{R}}}_{2}^{{{\text{Id}}}} \left( \omega \right)$, with $\omega - \omega_{c} = 0$, and can discriminate the path of photon D, according to the measurement outcome of QD5.

In the encoding process depicted in Fig. 6, an arbitrary-beam splitter (BS) is utilized to encode arbitrary quantum information onto the superposed state of the four-photon decoherence-free states [the output state $\left| {\psi_{8} } \right\rangle_{{{\text{ABCD}}}}$ (the superposition of logical qubits) from generation of decoherence-free states]. To determine the path of photon D, the interaction of the final gate, which utilizes the reflection operator $\hat{\text{R}}_{2}^{{{\text{Id}}}} \left( \omega \right)$ in Eq. 6, with $\omega - \omega_{c} = 0$, is performed between photon D and QD5, as described in Fig. 6.

In Fig. 6, an arbitrary-BS has the independent reflectivity and transmissivity, according to polarizations. The operation of an arbitrary-BS is given by

18 $$a_{{{\text{Down}}}}^{ + } \to \, \left( {\cos \phi } \right)a_{{{\text{Up}}}}^{ + } - \, \left( {\sin \phi } \right)a_{{{\text{Down}}}}^{ + } , a_{{{\text{Up}}}}^{ + } \to \, \left( {\sin \phi } \right)a_{{{\text{Up}}}}^{ + } + \, \left( {\cos \phi } \right)a_{{{\text{Down}}}}^{ + } ,$$

where $\cos \phi$ and $\sin \phi$ are the transmission and reflection coefficients of the arbitrary-BS^43,46,47,83^ for $\cos^{2} \phi + \sin^{2} \phi = 1$. Therefore, by controlling the experimental parameter $\phi$, we can generate the arbitrary encoding values (we want) onto four-photon decoherence-free states. After the transformed state $\left| {\psi_{8} } \right\rangle_{{{\text{ABCD}}}} = \left( {\left| {0_{{\text{ PL}}} } \right\rangle_{{{\text{ABCD}}}}^{1112} + \sqrt 3 \left| {1_{{\text{ PL}}} } \right\rangle_{{{\text{ABCD}}}}^{1111} } \right)/2$ by Feed-Forward (Eq. 17, results: $\left| { \pm_{{\text{e}}} } \right\rangle_{1} \left| { \pm_{{\text{e}}} } \right\rangle_{2} \left| { \pm_{{\text{e}}} } \right\rangle_{3} \left| { +_{{\text{e}}} } \right\rangle_{4}$ in Table 1), photon D in the state $\left| {\psi_{8} } \right\rangle_{{{\text{ABCD}}}}$ passes through the arbitrary-BS in Fig. 6. The arbitrary quantum information is then encoded as the state $\left| {\psi_{{\text{E}}} } \right\rangle_{{{\text{ABCD}}}}$, as follows:

19 $$\begin{aligned} \left| {\psi_{8} } \right\rangle_{{{\text{ABCD}}}}& {\mathop{\longrightarrow}\limits^{{\text{Arbitrary -}\text{ BS}}}} \\ \quad\left| {\psi_{{\text{E}}} } \right\rangle_{{{\text{ABCD}}}} & = \frac{1}{2}\left[ {\left( {\tau_{1} \left| {0_{{\text{ PL}}} } \right\rangle_{{{\text{ABCD}}}}^{1111} - \tau_{2} \sqrt 3 \left| {1_{{\text{ PL}}} } \right\rangle_{{{\text{ABCD}}}}^{1111} } \right) + \left( {\tau_{2} \left| {0_{{\text{ PL}}} } \right\rangle_{{{\text{ABCD}}}}^{1112} + \tau_{1} \sqrt 3 \left| {1_{{\text{ PL}}} } \right\rangle_{{{\text{ABCD}}}}^{1112} } \right)} \right] \\ & \equiv \frac{1}{\sqrt 2 }\left[ {\left( {\alpha_{1} \left| {0_{{\text{ PL}}} } \right\rangle_{{{\text{ABCD}}}}^{1111} + \beta_{1} \left| {1_{{\text{ PL}}} } \right\rangle_{{{\text{ABCD}}}}^{1111} } \right) + \left( {\alpha_{2} \left| {0_{{\text{ PL}}} } \right\rangle_{{{\text{ABCD}}}}^{1112} + \beta_{2} \left| {1_{{\text{ PL}}} } \right\rangle_{{{\text{ABCD}}}}^{1112} } \right)} \right], \\ \end{aligned}$$

where the specific transmission rate ($\tau_{1} \equiv \cos \phi$) and reflection rate ($\tau_{2} \equiv \sin \phi$) of the arbitrary-BS can be adjusted for our purposes (e.g., communication, information transfer, or computation) by controlling the experimental parameter $\phi$, as described in Eq. 18. For convenience, we define the arbitrary quantum information as $\left\{ {\alpha_{1} , \, \beta_{1} } \right\} \equiv \left\{ {\tau_{{1}} /\sqrt {\left| {\tau_{{1}} } \right|^{2} + 3\left| {\tau_{{2}} } \right|^{2} } , \, - \tau_{{2}} \sqrt 3 /\sqrt {\left| {\tau_{{1}} } \right|^{2} + 3\left| {\tau_{{2}} } \right|^{2} } } \right\}$ and $\left\{ {\alpha_{2} , \, \beta_{2} } \right\} \equiv \left\{ {\tau_{{2}} /\sqrt {\left| {\tau_{{2}} } \right|^{2} + 3\left| {\tau_{{1}} } \right|^{2} } , \, \tau_{{1}} \sqrt 3 /\sqrt {\left| {\tau_{{2}} } \right|^{2} + 3\left| {\tau_{{1}} } \right|^{2} } } \right\}$. As an additional example, (other measurement outcomes $\left| { \pm_{{\text{e}}} } \right\rangle_{1} \left| { \pm_{{\text{e}}} } \right\rangle_{2} \left| { \pm_{{\text{e}}} } \right\rangle_{3} \left| { -_{{\text{e}}} } \right\rangle_{4}$ in Table 1), if another output state $\left| {\psi^{\prime}_{8} } \right\rangle_{{{\text{ABCD}}}}$ is in the state $\left| {\psi^{\prime}_{8} } \right\rangle_{{{\text{ABCD}}}} = \left( {\left| {0_{{\text{ PL}}} } \right\rangle_{{{\text{ABCD}}}}^{1122} + \sqrt 3 \left| {1_{{\text{ PL}}} } \right\rangle_{{{\text{ABCD}}}}^{1121} } \right)/2$ from the generation of four-photon decoherence-free states, the arbitrary quantum information can be also encoded as the $\left| {\psi^{\prime}_{{\text{E}}} } \right\rangle_{{{\text{ABCD}}}}$ state, as follows:

20 $$\left| {\psi^{\prime}_{8} } \right\rangle_{{{\text{ABCD}}}} {\mathop{\longrightarrow}\limits^{{\text{Arbitrary-} \text{ BS}}}} \, \left| {\psi^{\prime}_{{\text{E}}} } \right\rangle_{{{\text{ABCD}}}} = \frac{1}{\sqrt 2 }\left[ {\left( {\alpha_{1} \left| {0_{{\text{ PL}}} } \right\rangle_{{{\text{ABCD}}}}^{1121} + \beta_{1} \left| {1_{{\text{ PL}}} } \right\rangle_{{{\text{ABCD}}}}^{1121} } \right) + \left( {\alpha_{2} \left| {0_{{\text{ PL}}} } \right\rangle_{{{\text{ABCD}}}}^{1122} + \beta_{2} \left| {1_{{\text{ PL}}} } \right\rangle_{{{\text{ABCD}}}}^{1122} } \right)} \right].$$

In Eqs. 19 and 20, the paths of the photon D are still split (are superposed in terms of paths: 1 and 2), despite the arbitrary information can be encoded onto the decoherence-free states (logical qubits). Thus, to merge the split paths, the final gate (an interaction between photon D and QD5 in Fig. 6) should be operated in the encoding process. Subsequently, after the operation of the final gate to the state $\left| {\psi_{{\text{E}}} } \right\rangle_{{{\text{ABCD}}}}$ in Eq. 19, the output state $\left| {\psi_{{\text{E}}} } \right\rangle_{{{\text{5ABCD}}}}^{{\text{i}}}$ (pre-measurement) is given by:

21 $$\begin{aligned} & \left| {\psi_{{\text{E}}} } \right\rangle_{{{\text{ABCD}}}} \otimes \left| { +_{{\text{e}}} } \right\rangle_{5} {\mathop{\longrightarrow}\limits^{{{\text{Final }}\;{\text{gate}}}}} \, \left| {\psi_{{\text{E}}} } \right\rangle_{{{\text{5ABCD}}}}^{{\text{i}}} \\&\quad = \frac{1}{\sqrt 2 }\left| { +_{{\text{e}}} } \right\rangle_{5} \otimes \left[ {\alpha_{1} \left| {0_{{\text{ PL}}} } \right\rangle_{{{\text{ABCD}}}}^{1111} + \beta_{1} \left| {1_{{\text{ PL}}} } \right\rangle_{{{\text{ABCD}}}}^{1111} } \right] \\ &\quad\quad+ \frac{1}{\sqrt 2 }\left| { -_{{\text{e}}} } \right\rangle_{5} \otimes \left[\vphantom{{+ \frac{{\beta_{2} }}{{\sqrt {12} }}\left( {2\left| {RRLL} \right\rangle - 2\left| {LLRR} \right\rangle + \left| {RLRL} \right\rangle - \left| {RLLR} \right\rangle + \left| {LRRL} \right\rangle - \left| {LRLR} \right\rangle } \right)_{{{\text{ABCD}}}}^{{{1112}}} } } {\frac{{\alpha_{2} }}{2}\left( { - \left| {RLRL} \right\rangle + \left| {RLLR} \right\rangle + \left| {LRRL} \right\rangle - \left| {LRLR} \right\rangle } \right)_{{{\text{ABCD}}}}^{{{1112}}}}\right. \\&\qquad\quad \left.{+ \frac{{\beta_{2} }}{{\sqrt {12} }}\left( {2\left| {RRLL} \right\rangle - 2\left| {LLRR} \right\rangle + \left| {RLRL} \right\rangle - \left| {RLLR} \right\rangle + \left| {LRRL} \right\rangle - \left| {LRLR} \right\rangle } \right)_{{{\text{ABCD}}}}^{{{1112}}} } \right] \\ \end{aligned}$$

where the prepared state of electron 5 in QD5 is in the state $\left| { +_{{\text{e}}} } \right\rangle_{{5}}$. According to the measurement outcomes of QD5, we can then acquire the final state of the arbitrary quantum information encoded onto the four-photon decoherence-free states (single logical qubit information), as follows:

22 $$\begin{aligned} & \left| {\psi_{{\text{E}}} } \right\rangle_{{{\text{5ABCD}}}}^{{\text{i}}} \, {\mathop{\longrightarrow}\limits^{{{\text{measurement}}}}} \, \left[ {{\text{result: }}\left| { +_{{\text{e}}} } \right\rangle_{{5}} } \right] \, {\mathop{\longrightarrow}\limits^{{{\text{Nothing}}}}} \, \left| {\psi_{{\text{E}}} } \right\rangle_{{{\text{ABCD}}}}^{{{\text{F(}} + )}} = \alpha_{1} \left| {0_{{\text{ PL}}} } \right\rangle_{{{\text{ABCD}}}}^{1111} + \beta_{1} \left| {1_{{\text{ PL}}} } \right\rangle_{{{\text{ABCD}}}}^{1111} , \\ & \left| {\psi_{{\text{E}}} } \right\rangle_{{{\text{5ABCD}}}}^{{\text{i}}} \, {\mathop{\longrightarrow}\limits^{{{\text{measurement}}}}} \, \left[ {{\text{result: }}\left| { -_{{\text{e}}} } \right\rangle_{{5}} } \right] \, {\mathop{\longrightarrow}\limits^{{\text{Feed}- \text{ Forward}}}} \, \left| {\psi_{{\text{E}}} } \right\rangle_{{{\text{ABCD}}}}^{{{\text{F(}} - )}} = \alpha_{2} \left| {0_{{\text{ PL}}} } \right\rangle_{{{\text{ABCD}}}}^{1112} + \beta_{2} \left| {1_{{\text{ PL}}} } \right\rangle_{{{\text{ABCD}}}}^{1112} , \\ \end{aligned}$$

where Feed-Forward [$\left| R \right\rangle$-phase flippers (RP)] is operated if the measurement outcome is in the state $\left| { -_{{\text{e}}} } \right\rangle_{{5}}$. Furthermore, for another state $\left| {\psi^{\prime}_{{\text{E}}} } \right\rangle_{{{\text{ABCD}}}}$ in Eq. 20, the final state (single logical qubit information) through the final gate can be obtained as:

23 $$\begin{aligned} & \left| {\psi^{\prime}_{{\text{E}}} } \right\rangle_{{{\text{5ABCD}}}}^{{\text{i}}} \, {\mathop{\longrightarrow}\limits^{{{\text{measurement}}}}} \, \left[ {{\text{result: }}\left| { +_{{\text{e}}} } \right\rangle_{{5}} } \right] \, {\mathop{\longrightarrow}\limits^{{{\text{Nothing}}}}} \, \left| {\psi^{\prime}_{{\text{E}}} } \right\rangle_{{{\text{ABCD}}}}^{{{\text{F(}} + )}} = \alpha_{1} \left| {0_{{\text{ PL}}} } \right\rangle_{{{\text{ABCD}}}}^{1121} + \beta_{1} \left| {1_{{\text{ PL}}} } \right\rangle_{{{\text{ABCD}}}}^{1121} , \\ & \left| {\psi^{\prime}_{{\text{E}}} } \right\rangle_{{{\text{5ABCD}}}}^{{\text{i}}} \, {\mathop{\longrightarrow}\limits^{{{\text{measurement}}}}} \, \left[ {{\text{result: }}\left| { -_{{\text{e}}} } \right\rangle_{{5}} } \right] \, {\mathop{\longrightarrow}\limits^{{\text{Feed-}\text{ Forward}}}} \, \left| {\psi^{\prime}_{{\text{E}}} } \right\rangle_{{{\text{ABCD}}}}^{{{\text{F(}} - )}} = \alpha_{2} \left| {0_{{\text{ PL}}} } \right\rangle_{{{\text{ABCD}}}}^{1122} + \beta_{2} \left| {1_{{\text{ PL}}} } \right\rangle_{{{\text{ABCD}}}}^{1122} , \\ \end{aligned}$$

where the final state, $\left| {\psi^{\prime}_{{\text{E}}} } \right\rangle_{{{\text{ABCD}}}}^{{{\text{F(}} + )}}$ or $\left| {\psi^{\prime}_{{\text{E}}} } \right\rangle_{{{\text{ABCD}}}}^{{{\text{F(}} - )}}$, shows another path, 2, for photon C, compared with Eq. 22.

We have designed a scheme to encode arbitrary quantum information onto four-photon decoherence-free states (single logical qubit information) using QD-cavity systems and linearly optical devices for immunity against collective decoherence. For the experimental implementation of our scheme, we analyze the interactions between a photon and an excess electron in a QD, within a single-sided cavity.

## Analysis of the interaction between a photon and electron in the quantum dot under vacuum noise and sideband leakage

For a reliable performance of the encoding scheme (single logical qubit information) in Sect. 2.3, the critical components are the QD-cavity systems, which can perform the reflection operators, ${\hat{\text{R}}}_{1}^{{{\text{Id}}}}$ [$\omega - \omega_{c} = \kappa /2$] and ${\hat{\text{R}}}_{2}^{{{\text{Id}}}}$ [$\omega - \omega_{c} = 0$], to induce differences in the reflectances $\left[ { \, \left| {R_{{\text{h}}} } \right|, \, \left| {R_{{0}} } \right| \, } \right]$ and phase shifts $\left[ {\varphi_{{{\text{Rh}}}} , \, \varphi_{{{\text{R0}}}} } \right]$ of the reflected photon, according to the hot or cold cavity. Therefore, the analysis of the interaction between a photon and an electron spin state in the QD is required to quantify the efficiency and reliability of the QD-cavity system under vacuum noise $N\left( \omega \right)$, for the operation of the QD-dipole, and leaky modes $S\left( \omega \right)$ (sideband leakage and absorption)^11,20,22,54–56,84,85^. To figure out the affections of the vacuum noise $N\left( \omega \right)$ and leaky modes $S\left( \omega \right)$ in the QD-cavity system, we can calculate the quantum Langevin equations of a cavity field operator $\hat{a}$, a dipole operator $\hat{\sigma }_{ - }$ of ${\text{X}}^{ - }$, and the input–output relations with vacuum noise $N\left( \omega \right)$ and leaky modes $S\left( \omega \right)$ from the Jaynes-Cummings Hamiltonian $H_{{{\text{JC}}}}$^11,20,22,54–56,84,85^, as follows:

24 $$\begin{aligned} & \frac{{d\hat{a}}}{dt} = - \frac{i}{\hbar }\left[ {\hat{a}, \, H_{{{\text{JC}}}} } \right] = - \left[ {i\left( {\omega_{c} - \omega } \right) + \kappa /2 + \kappa_{s} /2} \right]\hat{a} - g\hat{\sigma }_{ - } - \sqrt \kappa \hat{b}_{{{\text{in}}}} - \sqrt {\kappa_{s} } \hat{S}_{{{\text{in}}}} , \\ & \frac{{d\hat{\sigma }_{ - } }}{dt} = - \frac{i}{\hbar }\left[ {\hat{\sigma }_{ - } , \, H_{{{\text{JC}}}} } \right] = \left[ {i\left( {\omega_{{{\text{X}}^{ - } }} - \omega } \right) + \gamma /2} \right]\hat{\sigma }_{ - } - g\hat{\sigma }_{Z} \hat{a} + \sqrt \gamma \hat{N}, \\ & \hat{b}_{{{\text{out}}}} = \hat{b}_{{{\text{in}}}} + \sqrt \kappa \hat{a} \, , \quad \hat{S}_{{{\text{out}}}} = \hat{S}_{{{\text{in}}}} + \sqrt \kappa \hat{a} \, , \\ \end{aligned}$$

where $\hat{S}_{{{\text{in}}}}$ ($\hat{S}_{{{\text{out}}}}$) is the input (output) field operator from the leaky modes, due to sideband leakage and absorption in the cavity mode, and $\hat{N}$ is the vacuum noise operator for $\hat{\sigma }_{ - }$ of ${\text{X}}^{ - }$. In Sect. 2.2, we assumed the approximation of weak excitation with the ground state in the QD, $\left\langle {\hat{\sigma }_{Z} } \right\rangle = - 1$ (no saturation), for the steady state, $d\hat{\sigma }_{Z} /dt = 0$^11,20,22,54–56,84,85^. Therefore, we can calculate the noise $N_{{\text{h}}}$ ($N_{{0}}$) and leakage $S_{{\text{h}}}$ ($S_{{0}}$) coefficients of the hot (cold) cavity, with $\omega_{{{\text{X}}^{ - } }} = \omega_{c}$, as follows:

25 $$\begin{aligned} & \left[ {g \ne 0} \right]: \\ &N\left( \omega \right) = N_{{\text{h}}} \left( \omega \right) \equiv \left| {N_{{\text{h}}} \left( \omega \right)} \right|\exp \left[ {i\varphi_{{{\text{Nh}}}} \left( \omega \right)} \right] = \frac{{\sqrt {\gamma \kappa } g}}{{\left[ {i\left( {\omega_{c} - \omega } \right) + \gamma /2} \right]\left[ {i\left( {\omega_{c} - \omega } \right) + \kappa /2 + \kappa_{s} /2} \right] + g^{2} }}, \\ & S\left( \omega \right) = S_{{\text{h}}} \left( \omega \right) \equiv \left| {S_{{\text{h}}} \left( \omega \right)} \right|\exp \left[ {i\varphi_{{{\text{Sh}}}} \left( \omega \right)} \right] = \frac{{ - \sqrt {\kappa_{s} \kappa } \left[ {i\left( {\omega_{c} - \omega } \right) + \gamma /2} \right]}}{{\left[ {i\left( {\omega_{c} - \omega } \right) + \gamma /2} \right]\left[ {i\left( {\omega_{c} - \omega } \right) + \kappa /2 + \kappa_{s} /2} \right] + g^{2} }}, \\ & \left[ {g = 0} \right]: \\& N_{{0}} \left( \omega \right) \equiv \left| {N_{{0}} \left( \omega \right)} \right|\exp \left[ {i\varphi_{{{\text{N0}}}} \left( \omega \right)} \right] = 0 \, , \\ & S_{{0}} \left( \omega \right) \equiv \left| {S_{{0}} \left( \omega \right)} \right|\exp \left[ {i\varphi_{{{\text{S0}}}} \left( \omega \right)} \right] = \frac{{ - \sqrt {\kappa_{s} \kappa } }}{{i\left( {\omega_{c} - \omega } \right) + \kappa /2 + \kappa_{s} /2}}, \\ \end{aligned}$$

where the reflection coefficient $R_{{\text{h}}}$ ($R_{{0}}$) is expressed in Eq. 4 [$\left| {R_{{\text{h}}} } \right|$ ($\left| {R_{{0}} } \right|$): reflectance and $\varphi_{{{\text{Rh}}}}$ ($\varphi_{{{\text{R0}}}}$): phase shift] of the hot (cold) cavity. Additionally, we can obtain the noise rate $\left| {N_{{\text{h}}} } \right|$ ($\left| {N_{{0}} } \right|$) and phase shift $\varphi_{{{\text{Nh}}}}$ ($\varphi_{{{\text{N0}}}}$) from the vacuum noise and leakage rate $\left| {S_{{\text{h}}} } \right|$ ($\left| {S_{{0}} } \right|$) and phase shift $\varphi_{{{\text{Sh}}}}$ ($\varphi_{{{\text{S0}}}}$) from the leaky modes (sideband leakage and absorption) in the hot (cold) cavity. According to Eq. 25 (and the noise $N$ and leakage $S$ coefficients), the ideal reflection operator $\hat{\text{R}}\left( \omega \right)$, in Eq. 5, should be modified to a practical reflection operator ${\hat{\text{R}}}^{\Pr } \left( \omega \right)$, as follows:

26 $$\begin{aligned} & {\hat{\text{R}}}\left( \omega \right)\mathop \Rightarrow \limits^{{{\text{modification}}}} \,{\hat{\text{R}}}^{\Pr } \left( \omega \right)\\ & = \left[ { \, \left| {R_{{\text{h}}} \left( \omega \right)} \right|e^{{i\varphi_{{{\text{Rh}}}} \left( \omega \right)}} + \left| {N_{{\text{h}}} \left( \omega \right)} \right|e^{{i\varphi_{{{\text{Nh}}}} \left( \omega \right)}} + \left| {S_{{\text{h}}} \left( \omega \right)} \right|e^{{i\varphi_{{{\text{Sh}}}} \left( \omega \right)}} \, } \right]\left( {\left| R \right\rangle \left\langle R \right| \otimes \left| \downarrow \right\rangle \left\langle \downarrow \right| + \left| L \right\rangle \left\langle L \right| \otimes \left| \uparrow \right\rangle \left\langle \uparrow \right|} \right)\\& \quad+ \left[ { \, \left| {R_{{0}} \left( \omega \right)} \right|e^{{i\varphi_{{{\text{R0}}}} \left( \omega \right)}} + \left| {S_{{0}} \left( \omega \right)} \right|e^{{i\varphi_{{{\text{S0}}}} \left( \omega \right)}} \, } \right]\left( {\left| R \right\rangle \left\langle R \right| \otimes \left| \uparrow \right\rangle \left\langle \uparrow \right| + \left| L \right\rangle \left\langle L \right| \otimes \left| \downarrow \right\rangle \left\langle \downarrow \right|} \right), \\ \end{aligned}$$

where $N_{{0}} \left( \omega \right) \equiv \left| {N_{{0}} \left( \omega \right)} \right|\exp \left[ {i\varphi_{{{\text{N0}}}} \left( \omega \right)} \right] = 0$ (cold cavity: $g = 0$) from Eq. 25.

In Table 2, to analyze the affections of vacuum noise $$N\left( \omega \right)$$ and leaky modes $$S\left( \omega \right)$$ in the QD-cavity system, the values of the reflection $$R\left( \omega \right)$$, noise $$N\left( \omega \right)$$, and leakage $$S\left( \omega \right)$$ coefficients (of the hot and cold cavities) are listed from Eqs. 4 and 24, for different side-leakage rates $$\kappa_{s} /\kappa$$ (0.01, 1.00, and 2.00) and strong (weak) coupling strength $$g/\kappa = 2.4$$ ($$g/\kappa = 0.1$$), with a fixed decay rate, $$\gamma /\kappa = 0.1$$, and $$\omega_{{{\text{X}}^{ - } }} = \omega_{c}$$. Additionally, for the practical reflection operator $${\hat{\text{R}}}^{\Pr }$$ (QD1, QD2, and QD3: $$\omega - \omega_{c} = \kappa /2$$) and (QD4 and QD5: $$\omega - \omega_{c} = 0$$), we can confirm the values of the reflection $$R_{{\text{h}}} \, \left( {R_{{0}} } \right)$$, noise $$N_{{\text{h}}}$$, and leakage $$S_{{\text{h}}} \, \left( {S_{{0}} } \right)$$ coefficients in the hot (cold) cavity, where $$N_{{0}} = 0$$, from Eq. 25, as listed in Table 2. We can obtain the rates and phase shifts of the noise and leakage as $$\left| {N_{{\text{h}}} } \right| = \left| {S_{{\text{h}}} } \right| = \left| {S_{{0}} } \right| \approx 0$$, $$\left| {N_{{0}} } \right| = 0$$, and $$\varphi_{{{\text{Nh}}}} = \varphi_{{{\text{Sh}}}} = \varphi_{{{\text{S0}}}} \approx 0$$, $$\varphi_{{{\text{N0}}}} = 0$$ for both $$\omega - \omega_{c} = \kappa /2$$ (QD1, QD2, and QD3) and $$\omega - \omega_{c} = 0$$ (QD4 and QD5), when the experimental parameters exhibit a strong coupling strength, $$g/\kappa = 2.4$$, and an insignificantly small side-leakage rate, $$\kappa_{s} /\kappa = 0.01$$, in Table 2. This result indicates that the vacuum noise $$N\left( \omega \right)$$ for the QD-dipole operation and the leaky modes $$S\left( \omega \right)$$ (sideband leakage and absorption) for the cavity mode in the QD-cavity system can be ignored by choosing the parameters $$g/\kappa = 2.4$$ and $$\kappa_{s} \approx 0$$, with $$\gamma /\kappa = 0.1$$. Furthermore, in the case of these parameters, the values of the reflectances $$\left\{ {\left| {R_{{\text{h}}} } \right|, \, \left| {R_{{0}} } \right|} \right\}$$ and phase shifts $$\left\{ {\varphi_{{{\text{Rh}}}} , \, \varphi_{{{\text{R0}}}} } \right\}$$ approach the values of the ideal reflection operators $$\omega - \omega_{c} = \kappa /2 \, \to \, \hat{\text{R}}_{1}^{{{\text{Id}}}}$$$$\left( {\omega - \omega_{c} = 0 \, \to \, \hat{\text{R}}_{2}^{{{\text{Id}}}} } \right)$$ of Eq. 6, such that $$\left| {R_{{\text{h}}} } \right| \approx \left| {R_{{0}} } \right| \approx 0.99$$$$\left( {\left| {R_{{\text{h}}} } \right| \approx 0.99, \, \left| {R_{{0}} } \right| \approx 0.98} \right)$$ and $$\varphi_{{{\text{Rh}}}} \approx 0.09, \, \varphi_{{{\text{R0}}}} \approx - 1.57$$$$\left( {\varphi_{{{\text{Rh}}}} \approx 0.00, \, \varphi_{{{\text{R0}}}} \approx 3.14} \right)$$.

**Table 2:** In the cases of frequency detunings, where $\omega - \omega_{c} = \kappa /2$ [QD1, QD2, and QD3] and $\omega - \omega_{c} = 0$ [QD4 and QD5], the values of the reflection $R_{{\text{h}}} \left( {R_{{0}} } \right)$, noise $N_{{\text{h}}}$, and leakage $S_{{\text{h}}} \left( {S_{{0}} } \right)$ coefficients in hot (cold) cavity were calculated for the differences in the side-leakage rate $\kappa_{s} {/}\kappa$ and the coupling strength $g{/}\kappa$, with fixed parameters $\gamma {/}\kappa = 0.1$ and $\omega_{{{\text{X}}^{ - } }} = \omega_{c}$.

$\omega - \omega_{c}$	$\gamma /\kappa = 0.1$		Reflection, noise, and leakage coefficients				
			Hot cavity			Cold cavity	
	$g/\kappa$	$\kappa_{s} /\kappa$	$R_{{\text{h}}} \left( \omega \right)$	$N_{{\text{h}}} \left( \omega \right)$	$S_{{\text{h}}} \left( \omega \right)$	$R_{{0}} \left( \omega \right)$	$S_{{0}} \left( \omega \right)$
$\kappa /2$	2.4	0.01	0.99 + 0.09i	0.14 + 0.01i	0.00 + 0.01i	0.00–0.99i	− 0.10–0.10i
		1.00	0.98 + 0.09i	0.14 + 0.01i	− 0.02 + 0.09i	0.20–0.40i	− 0.80–0.40i
		2.00	0.98 + 0.09i	0.13 + 0.02i	− 0.03 + 0.12i	0.40–0.20i	− 0.85–0.28i
	0.1	0.01	− 0.04–0.98i	− 0.05 + 0.07i	− 0.10–0.10i	0.00–0.99i	− 0.10–0.10i
		1.00	0.19–0.39i	− 0.02 + 0.05i	− 0.81–0.39i	0.20–0.40i	− 0.80–0.40i
		2.00	0.40–0.19i	− 0.01 + 0.04i	− 0.85–0.27i	0.40–0.20i	− 0.85–0.28i
$0$	2.4	0.01	0.9914	0.1312	− 0.0008	− 0.9802	− 0.1980
		1.00	0.9914	0.1306	− 0.0086	0.0000	− 1.0000
		2.00	0.9914	0.1301	− 0.0121	0.3333	− 0.9428
	0.1	0.01	− 0.4184	0.8971	− 0.1418	− 0.9802	− 0.1980
		1.00	0.1667	0.5270	− 0.8333	0.0000	− 1.0000
		2.00	0.4118	0.3720	− 0.8319	0.3333	− 0.9428

Furthermore, we can quantify the efficiency and reliability of the QD-cavity system from the calculation of average of fidelities (AoFs), AoF_1_ ($\omega - \omega_{c} = \kappa /2$: QD1, QD2, QD3) and AoF_2_ ($\omega - \omega_{c} = 0$: QD4, QD5), using the vacuum noise $N\left( \omega \right)$ and the leaky modes $S\left( \omega \right)$. For example, let us assume that the arbitrary input state of photon-electron is $\left( {\cos \vartheta \left| R \right\rangle + \sin \vartheta \left| L \right\rangle } \right) \otimes \left( {\cos \xi \left| \uparrow \right\rangle + \sin \xi \left| \downarrow \right\rangle } \right)$ for $\cos^{2} \vartheta + \sin^{2} \vartheta = \cos^{2} \xi + \sin^{2} \xi = 1$. If we can consider the ideal case (no vacuum noise and leaky mode), a strong coupling strength $g \gg \left( {\kappa , \, \gamma } \right)$, and a small side-leakage rate $\kappa_{s} \ll \kappa$ with a small $\gamma /\kappa$, the ideal output states, $\left| {\upphi _{ \, 1}^{{{\text{Id}}}} } \right\rangle$ and $\left| {\upphi _{ \, 2}^{{{\text{Id}}}} } \right\rangle$, from the reflection operators, $\hat{\text{R}}_{1}^{{{\text{Id}}}}$ and $\hat{\text{R}}_{2}^{{{\text{Id}}}}$, are given by:

27 $$\begin{aligned} & \left[ {\omega - \omega_{c} = \kappa /2} \right] \, \Rightarrow \\& \left| {\upphi _{ \, 1}^{{{\text{Id}}}} } \right\rangle = \left( {\cos \vartheta \sin \xi \left| R \right\rangle \left| \downarrow \right\rangle + \sin \vartheta \cos \xi \left| L \right\rangle \left| \uparrow \right\rangle } \right) \, - \, i\left( {\cos \vartheta \cos \xi \left| R \right\rangle \left| \uparrow \right\rangle + \sin \vartheta \sin \xi \left| L \right\rangle \left| \downarrow \right\rangle } \right), \\ & \left[ {\omega - \omega_{c} = 0} \right] \, \Rightarrow \\& \left| {\upphi _{ \, 2}^{{{\text{Id}}}} } \right\rangle = \left( {\cos \vartheta \sin \xi \left| R \right\rangle \left| \downarrow \right\rangle + \sin \vartheta \cos \xi \left| L \right\rangle \left| \uparrow \right\rangle } \right) \, - \, \left( {\cos \vartheta \cos \xi \left| R \right\rangle \left| \uparrow \right\rangle + \sin \vartheta \sin \xi \left| L \right\rangle \left| \downarrow \right\rangle } \right). \\ \end{aligned}$$

While, the practical output state $\left| {\upphi ^{{\Pr}} } \right\rangle$ from the practical reflection operator $\hat{\text{R}}^{\Pr }$ (including the vacuum noise and leaky mode), in Eq. 26, is expressed as:

28 $$ \left| {\upphi ^{{\Pr}} } \right\rangle = \frac{1}{{\sqrt {\text{N}} }}\left[ {\left( {R_{{\text{h}}} + N_{{\text{h}}} + S_{{\text{h}}} } \right)\left( {\cos \vartheta \sin \xi \left| R \right\rangle \left| \downarrow \right\rangle + \sin \vartheta \cos \xi \left| L \right\rangle \left| \uparrow \right\rangle } \right) + \left( {R_{{0}} + S_{{0}} } \right)\left( {\cos \vartheta \cos \xi \left| R \right\rangle \left| \uparrow \right\rangle + \sin \vartheta \sin \xi \left| L \right\rangle \left| \downarrow \right\rangle } \right)} \right], $$

where ${\text{N}} \equiv \left| {R_{{\text{h}}} + N_{{\text{h}}} + S_{{\text{h}}} } \right|^{2} \left( {\cos^{2} \vartheta \sin^{2} \xi + \sin^{2} \vartheta \cos^{2} \xi } \right) + \left| {R_{{0}} + S_{{0}} } \right|^{2} \left( {\cos^{2} \vartheta \cos^{2} \xi + \sin^{2} \vartheta \sin^{2} \xi } \right)$. From these results, we can calculate the two AoF_1_ (QD1, QD2, and QD3) and AoF_2_ ($\omega - \omega_{c} = 0$: QD4, QD5), between the ideal output states, $\left| {\upphi _{ \, 1}^{{{\text{Id}}}} } \right\rangle$ and $\left| {\upphi _{ \, 2}^{{{\text{Id}}}} } \right\rangle$ (Eq. 27) and the practical output state $\left| {\upphi ^{{\Pr}} } \right\rangle$ (Eq. 28), to quantify the efficiency and reliability of the QD-cavity system, as follows:

29 $$ {\text{AoF}}_{1} = \frac{1}{{4\pi^{2} }}\int_{0}^{2\pi } {\int_{0}^{2\pi } {\left| {\sqrt {\left\langle {{\upphi _{{ 1}}^{{{\text{Id}}}} }} \mathrel{\left | {\vphantom {{\upphi _{{ 1}}^{{{\text{Id}}}} } {\upphi ^{{\Pr}} }}} \right. \kern-\nulldelimiterspace} {{\upphi ^{{\Pr}} }} \right\rangle \left\langle {{\upphi ^{{\Pr}} }} \mathrel{\left | {\vphantom {{\upphi ^{{\Pr}} } {\upphi _{{ 1}}^{{{\text{Id}}}} }}} \right. \kern-\nulldelimiterspace} {{\upphi _{{ 1}}^{{{\text{Id}}}} }} \right\rangle } } \right|d\vartheta d\xi } } ,{\text{ AoF}}_{2} = \frac{1}{{4\pi^{2} }}\int_{0}^{2\pi } {\int_{0}^{2\pi } {\left| {\sqrt {\left\langle {{\upphi _{{ 2}}^{{{\text{Id}}}} }} \mathrel{\left | {\vphantom {{\upphi _{{ 2}}^{{{\text{Id}}}} } {\upphi ^{{\Pr}} }}} \right. \kern-\nulldelimiterspace} {{\upphi ^{{\Pr}} }} \right\rangle \left\langle {{\upphi ^{{\Pr}} }} \mathrel{\left | {\vphantom {{\upphi ^{{\Pr}} } {\upphi _{{ 2}}^{{{\text{Id}}}} }}} \right. \kern-\nulldelimiterspace} {{\upphi _{{ 2}}^{{{\text{Id}}}} }} \right\rangle } } \right|d\vartheta d\xi } } . $$

**Figure 7:**
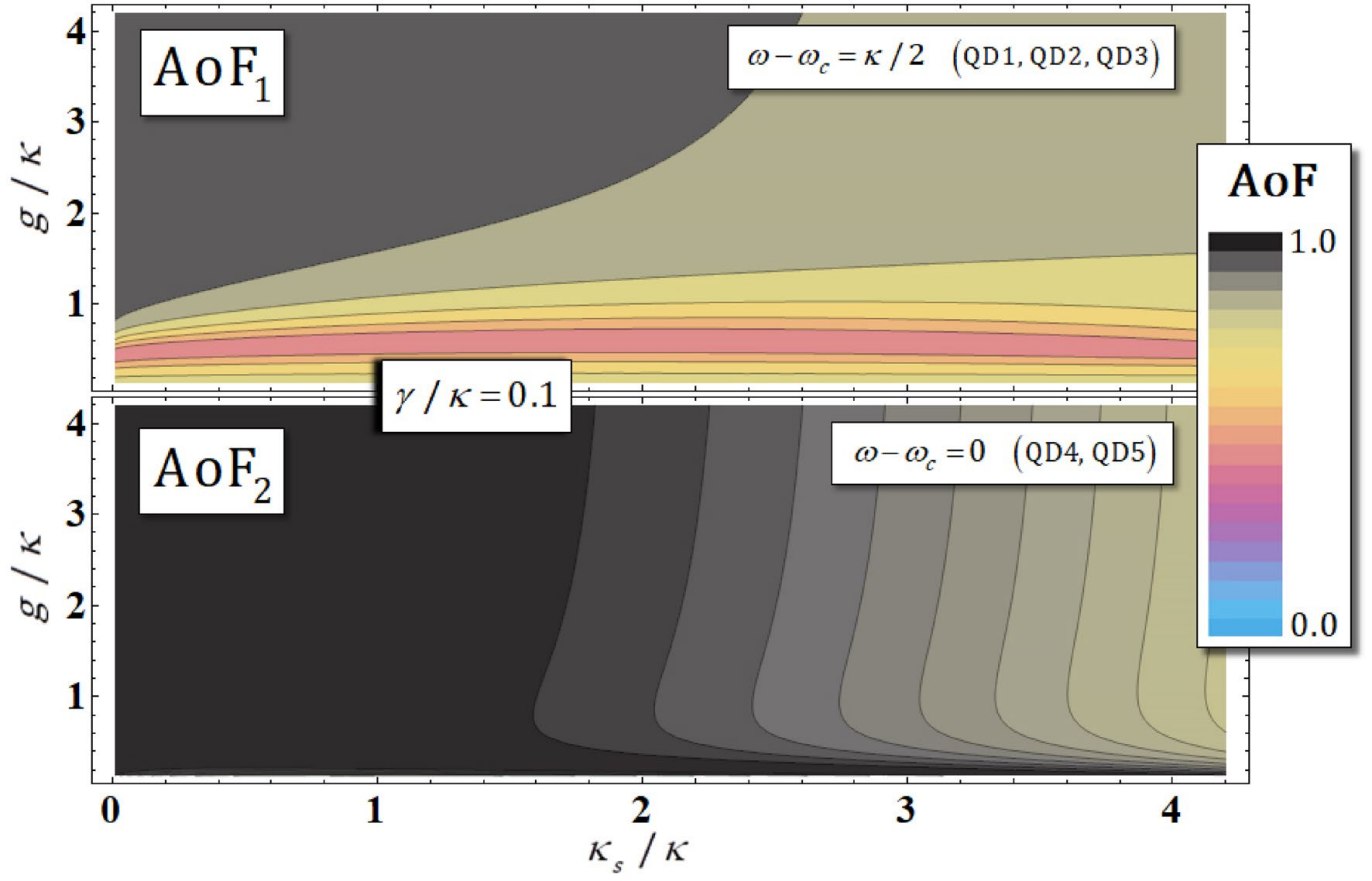
This graph shows the distributions of AoF_1_ (QD1, QD2, QD3: $\omega - \omega_{c} = \kappa /2$) and AoF_2_ (QD4, QD5: $\omega - \omega_{c} = 0$) of the output states for the side-leakage rate $\kappa_{s} /\kappa$ and coupling strength $g/\kappa$, with $\gamma /\kappa = 0.1$ and $\omega_{{{\text{X}}^{ - } }} = \omega_{c}$ under vacuum noise $N\left( \omega \right)$, for the operation of the QD-dipole, and leaky modes $S\left( \omega \right)$ (sideband leakage and absorption).

In Fig. 7, we depict the distributions of the AoF_1_ and AoF_2_, represented by differences in $\kappa_{s} /\kappa$ and $g/\kappa$ with $\gamma /\kappa = 0.1$.

We can also calculate the exact values of AoF_1_According to these distributions, we can confirm that both AoF_1_ and AoF_2_ tend to 1 (shown in red), as described in Fig. 7, when the parameters adhere to the following conditions: a strong coupling strength $g \gg \left( {\kappa , \, \gamma } \right)$ and a small side-leakage rate $\kappa_{s}$ ($\kappa_{s} \ll \kappa$), with a small $\gamma /\kappa$, with the effect under the affections of vacuum noise $N\left( \omega \right)$ and the leaky modes *S(ω*).

AoF_1_ and AoF_2_ to quantify the efficiency and performance of the QD-cavity systems via Eq. 29, as listed in Table 3. As shown in Table 3, as the magnitude of the coupling strength $g/\kappa$ increases and the side-leakage rate $\kappa_{s} /\kappa$ decreases in the QD-cavity systems (QD1, QD2, QD3: $\omega - \omega_{c} = \kappa /2$, QD4, QD5: $\omega - \omega_{c} = 0$), we can conclude that the influences of the vacuum noise $N\left( \omega \right)$ and leaky modes $S\left( \omega \right)$ (sideband leakage and absorption) can be ignored to obtain high efficiency and reliability of the QD-cavity systems (AoF_1_, AoF_2_ $\rightarrow$ 1).

**Table 3:** The values of AoF_1_ and AoF_2_ between Eq. 27 (the ideal output states from ${\hat{\text{R}}}_{1}^{{{\text{Id}}}}$ and ${\hat{\text{R}}}_{2}^{{{\text{Id}}}}$) and Eq. 28 (the practical output state from ${\hat{\text{R}}}^{{\Pr}}$) are listed. The values were calculated using Eq. 29, with consideration to the coupling strength $g/\kappa$ and the side-leakage rate $\kappa_{s} /\kappa$, with $\gamma /\kappa = 0.1$ and $\omega_{{{\text{X}}^{ - } }} = \omega_{c}$.

$\gamma /\kappa = 0.1$	$\kappa_{s} /\kappa$	AoF_1_ $\left( {\omega - \omega_{c} = \kappa /2} \right)$	AoF_2_ $\left( {\omega - \omega_{c} = 0} \right)$
$g/\kappa = 2.4$	0.01	0.9967	0.9997
	1.00	0.9381	0.9989
	2.00	0.8983	0.9688
$g/\kappa = 0.1$	0.01	0.7598	0.8906
	1.00	0.7652	0.5816
	2.00	0.7654	0.6163

Thus, we can demonstrate the method to experimentally implement QD-cavity systems with high efficiency and reliability, under the vacuum noise and leaky modes, if we consider a strong coupling strength $g \gg \left( {\kappa , \, \gamma } \right)$ and a small side-leakage rate $\kappa_{s} \ll \kappa$, using our analysis of the practical reflection operator $\hat{\text{R}}^{\Pr } \left( \omega \right)$ in Eq. 26 and the fidelities expressed in Eq. 29, Fig. 7, and Table 3.

## Conclusions

In various quantum information processing schemes, quantum entanglement (main resource) can be easily attenuated by the influence of the environment-system. To solve this issue, the concept of a decoherence-free subspace^32–35,43–45,50–52,59–61^ has been widely employed in quantum communications^86–89^ and quantum computations^90–92^ for robustness against collective decoherence^32–34^. In this paper, we proposed the encoding scheme, which consisted of the generation of four-photon decoherence-free states, and the encoding process to encode arbitrary quantum information onto logical qubits, using QD-cavity systems for single logical qubit information. Our work was motivated from the previous schemes^21,43,52^, which utilized XKNLs, in the generation of decoherence-free states, and encoding a quantum information. From the comparison with the previous works, we can demonstrate the advantages of our scheme, as follows: (1) In Refs.^21,43^, they designed to generate three-qubit decoherence-free states, and encode an arbitrary quantum information, but logical three-qubit^21,22,36–43,50,60,93^ could provide only the minimal immunity against collective decoherence. By this limited effect, the extending dimension of subsystem is obviously required. Therefore, our scheme, which utilized the QD-cavity systems, can accomplish to enhance the immunity by increasing the number of qubit (four-photon decoherence-free states). (2) In Refs.^43,52^, they overlooked the affections of decoherence effect^8,9,12–14,21^ in controlled gates using XKNLs. In practice, when implementing controlled gates by XKNLs, the decoherence effect induced by photon loss and dephasing is inevitable. Therefore, for our scheme, we employed the QD-cavity systems, which can well isolate quantum information from the environment, to obtain the long coherence time^58,62–67,76^ without decoherence effect.

Furthermore, in the case of four-qubit decoherence-free state, when to utilize SPDC^46,47^ or source of entangled state^48,49^ with linearly optical devices, the essential requirement is the preparation of entangled states beforehand. This means that the reliable performance of all procedures depend on the source of entangled state in spite of utilizing linearly optical device with ease. Also, in the previous schemes using cavity-QED^44,51^ or XKNLs^52,53^, these works ignored the vacuum noise in QD dipole operation and sideband leakage in cavity mode^44,51^, and the decoherence effect in nonlinearly optical gate^52,53^.

Compared with the previous works, our scheme can directly produce the correlations for entanglement by the QD-cavity systems without source of entangled state. Moreover, from our analysis of the influences of vacuum noise and leaky mode, we can support the experimental conditions to acquire high efficiency and reliability of the QD-cavity systems. Therefore, our scheme (encoding single logical qubit information onto four-photon decoherence-free states) can be applied to quantum information processing schemes to improve reliability when these schemes are operated under the collective decoherence, induced by the identical decoherence (dispersive energy or rotation noise) of each qubit in the system.

Moreover, because our scheme utilized the QDs within single-sided cavities, as the ancillary systems, the QD-cavity systems (QD1, QD2, QD3, QD4, and QD5) should be feasibly implemented. We demonstrated the conditions (strong coupling strength, $g \gg \left( {\kappa , \, \gamma } \right)$, and small side-leakage rate, $\kappa_{s} \ll \kappa$) acquired from our analysis, to obtain high fidelities, ${\text{F}}_{1}$ and ${\text{F}}_{2}$, of the QD-cavity system, which indicate high efficiency and reliability under the effect of vacuum noise $N\left( \omega \right)$ and leaky modes $S\left( \omega \right)$ (sideband leakage and absorption). Many other studies have been done to achieve the experimental conditions indicated in our analysis. A strong coupling strength of $g/\left( {\kappa + \kappa_{s} } \right) \approx 2.4$, for ${\text{Q}} = 40000$, was achieved by Hennessy et al.^94^. Arnold et al.^95^ enhanced the quality factor, ${\text{Q}} = 215000 \, \left( {\kappa \approx 6.2 \, \mu {\text{eV}}} \right)$, for a small side-leakage rate. In Ref.^96^, the side-leakage rate $\kappa_{s} /\kappa$ was reduced in an optical cavity (${\text{In}}_{0.6} {\text{Ga}}_{0.6} {\text{As}}$), with $g/\left( {\kappa + \kappa_{s} } \right) \approx 2.4$, for ${\text{Q}} = 40000$, using two methods (the etching process and by improving the sample growth).

Thus, we have proposed that the encoding scheme for single logical qubit information onto four-photon decoherence-free states, to prevent collective decoherence, can be experimentally implemented using QD-cavity systems. Moreover, through our analysis, high efficiency and reliability of the QD-cavity systems can be accomplished using a strong coupling strength and a small side-leakage rate, under the effect of vacuum noise and leaky modes.
